# Genome characterisation and comparative analysis of *Schaalia dentiphila* sp. nov. and its subspecies, *S. dentiphila* subsp*. denticola* subsp. nov., from the human oral cavity

**DOI:** 10.1186/s12866-024-03346-w

**Published:** 2024-05-28

**Authors:** Xuechen Tian, Wee Fei Aaron Teo, Yixin Yang, Linyinxue Dong, Aloysius Wong, Li Chen, Halah Ahmed, Siew Woh Choo, Nicholas S. Jakubovics, Geok Yuan Annie Tan

**Affiliations:** 1 Institute of Biological Sciences, Faculty of Science, Universiti Malaya, Kuala Lumpur, 50603 Malaysia; 2 College of Science, Mathematics and Technology, Wenzhou-Kean University, 88 Daxue Road, Ouhai, Wenzhou, Zhejiang Province 325060 China; 3 Wenzhou Municipal Key Laboratory for Applied Biomedical and Biopharmaceutical Informatics, Wenzhou-Kean University, Ouhai, Wenzhou, Zhejiang Province, 325060 China; 4 Zhejiang Bioinformatics International Science and Technology Cooperation Center, Wenzhou-Kean University, Ouhai, Wenzhou, Zhejiang Province, 325060 China; 5 Dorothy and George Hennings College of Science, Mathematics and Technology, Kean University, 1000 Morris Ave, Union, NJ 07083 USA; 6 School of Dental Sciences, Faculty of Medical Sciences, Newcastle University, Framlington Place, Newcastle Upon Tyne, NE2 4BW UK

**Keywords:** *Schaalia dentiphila*, *Schaalia dentiphila* subsp *denticola*, Novel species, Genome analysis, Taxonomy, Oral cavity

## Abstract

**Background:**

*Schaalia* species are primarily found among the oral microbiota of humans and other animals. They have been associated with various infections through their involvement in biofilm formation, modulation of host responses, and interaction with other microorganisms. In this study, two strains previously indicated as *Actinomyces* spp. were found to be novel members of the genus *Schaalia* based on their whole genome sequences.

**Results:**

Whole-genome sequencing revealed both strains with a genome size of 2.3 Mbp and GC contents of 65.5%. Phylogenetics analysis for taxonomic placement revealed strains NCTC 9931 and C24 as distinct species within the genus *Schaalia*. Overall genome-relatedness indices including digital DNA-DNA hybridization (dDDH), and average nucleotide/amino acid identity (ANI/AAI) confirmed both strains as distinct species, with values below the species boundary thresholds (dDDH < 70%, and ANI and AAI < 95%) when compared to nearest type strain *Schaalia odontolytica* NCTC 9935^ T^. Pangenome and orthologous analyses highlighted their differences in gene properties and biological functions compared to existing type strains. Additionally, the identification of genomic islands (GIs) and virulence-associated factors indicated their genetic diversity and potential adaptive capabilities, as well as potential implications for human health. Notably, CRISPR-Cas systems in strain NCTC 9931 underscore its adaptive immune mechanisms compared to strain C24.

**Conclusions:**

Based on these findings, strain NCTC 9931^T^ (= ATCC 17982^T^ = DSM 43331^T^ = CIP 104728^T^ = CCUG 18309^T^ = NCTC 14978^T^ = CGMCC 1.90328^T^) represents a novel species, for which the name *Schaalia dentiphila* subsp*. dentiphila* sp. nov. subsp. nov. is proposed, while strain C24^T^ (= NCTC 14980^T^ = CGMCC 1.90329^T^) represents a distinct novel subspecies, for which the name *Schaalia dentiphila* subsp*. denticola*. subsp. nov. is proposed. This study enriches our understanding of the genomic diversity of *Schaalia* species and paves the way for further investigations into their roles in oral health.

**Significance:**

This research reveals two *Schaalia* strains, NCTC 9931^ T^ and C24^T^, as novel entities with distinct genomic features. Expanding the taxonomic framework of the genus *Schaalia*, this study offers a critical resource for probing the metabolic intricacies and resistance patterns of these bacteria. This work stands as a cornerstone for microbial taxonomy, paving the way for significant advances in clinical diagnostics.

**Supplementary Information:**

The online version contains supplementary material available at 10.1186/s12866-024-03346-w.

## Introduction

*Schaalia* bacteria are Gram-strain positive and aerobic to facultatively anaerobic bacteria with morphology ranging from straight to slightly curved rods, with certain strains exhibiting branching patterns [1]. Members of the genus *Schaalia* predominantly inhabit the oral microbiota of humans and other animals and have been associated with certain human diseases. For instance, *Schaalia odontolytica* (formerly known as *Actinomyces odontolyticus*) is a species linked to oral infections [2, 3] and *Schaalia turicensis* was isolated from a surgical biopsy [4]. *Schaalia* species, along with others, play a pivotal role in maintaining oral health through their ability to form biofilm, modulate host responses, and interact with other microbial residents [3, 5].

There are currently 12 species with correct and validly published names within the genus *Schaalia* (https://lpsn.dsmz.de/genus/schaalia) [6]. However, fewer than ten genome assemblies for type strains of these species are publicly available within the National Center of Biotechnology Information (NCBI) database [7]. Additionally, only 66 search results were associated with the “*Schaalia”* term in the PubMed database (Accessed on April 15, 2024). This limited number of search results can be attributed to the fact that much of the research relevant to *Schaalia* was previously published under its former name, *Actinomyces*. However, most of the previous studies have focused on aspects such as biofilm formation and interactions with other bacteria, with less emphasis on the classification and systematic genomic analysis of new species under the *Schaalia* genus. This historical context of nomenclature change contributes to the apparent scarcity of data specifically labeled under the name *Schaalia*, highlighting the need for updated research and classification in this area.

In our comparative genomics study on oral *Actinomyces*, a few strains that were initially classified as *Actinomyces* showed significant genomic differences from typical *Actinomyces* strains, in particular strains previously reported as *Actinomyces odontolyticus* NCTC 9931 and *Actinomyces odontolyticus* C24 [8]. Strain C24, originally isolated from root surface caries of supragingival plaque in Papua New Guinea indigenes [9], was transferred to Newcastle University in 1993 by Prof. Dr. Roy R.B. Russell from the University of Sydney, Australia, and strain NCTC 9931 was used as one of the reference strains. It is important to note that biochemical tests within the API system offer only tentative species identification and whole genome sequencing can provide better taxonomic identity with comprehensive genomic profile.

In this study, strain NCTC 9931 was found to represent a novel *Schaalia* species, for which the name *Schaalia dentiphila* sp. nov. is proposed. Additionally, strain C24 which showed highly similar yet distinct genomic properties when compared to strain NCTC 9931, is proposed as a subspecies of *Schaalia dentiphila*. This research provides valuable insights into the diversity and genome characteristics of this important bacterial genus associated with human oral health and sets the stage for further investigations.

## Materials and methods

### Strains preparation

Strains are currently maintained in the laboratory of Prof. Nicholas S. Jakubovics at Newcastle University, United Kingdom, and their subcultures have been deposited in the collections of the National Collection of Type Cultures (NCTC), UK, and China General Microbiological Culture Collection Center (CGMCC), China. For routine cultivation, the strains were grown in BHYE broth, composed of 37 g/L of brain heart infusion and 5 g/L of yeast extract, under an anaerobic atmosphere at 37 °C. For long-term storage, strains were preserved at -80 °C in a broth solution containing 40% (v/v) glycerol.

### Genomic DNA extraction and library preparation for sequencing

The MasterPure™ Gram Positive DNA Purification Kit was used to extract the total DNA from bacterial cells following the manufacturer’s instructions. The quantification of DNA was determined using a NanoDrop spectrophotometer (Thermo Scientific), and DNA integrity and purity were verified using 1% (w/v) agarose gel electrophoresis. The genomic DNA (1 μg) was used for library construction by random fragmentation of the DNA using Covaris S2 for 120 s at 5.5—6.0 °C. The DNA fragments were then selected with an average size of 200–400 base pairs using magnetic beads and quantified using a Qubit fluorometer (Thermo Fisher Scientific). These fragments underwent end-repair and 3' adenylation using T4 DNA polymerase, Klenow DNA polymerase, and T4 polynucleotide kinase. Adapter sequences were subsequently ligated to the blunt ends of the 3' adenylated fragments. The ligated products were amplified by PCR. Following purification, the double-stranded PCR products were heat-denatured and circularized using the split oligo sequence. The final library was then prepared from the single-strand circular DNA and sequenced on the Illumina HiSeq X Ten platform with PE151 (paired-end) sequencing, following the manufacturer's recommended protocols (Illumina). The resulting raw sequence data were generated in fastq format.

### Data preprocessing, genome assembly and annotation

The generated raw data sequencing underwent preprocessing, which involved filtering out lower quality data including adapter and duplication contamination, using PRINSEQ lite software [10]. Bactopia pipeline (v 1.7.1) [11] was employed to assemble clean reads into genomes. The assembled quality was evaluated via the Quality Assessment Tool for Genome Assemblies [12]. Additionally, the genome completeness assessment was conducted through the CheckM (v 1.2.2) tool [13]. To further verify the genome assembly quality, a more accurate genome assembly assessment online interface, gVolante2 [14] was used based on the BUSCO v5 tool [15]. All assembled genomes with high quality were annotated using the Rapid Annotation using Subsystem Technology (RAST) webserver (https://rast.nmpdr.org) [16]. For consistency, publicly available type strain genomes of the *Schaalia* species with validly published name, were downloaded from the NCBI RefSeq database [17], and subjected to identical annotation using RAST.

### 16S rRNA phylogeny and multilocus sequence analysis

To determine the preliminary species identification, the 16S rRNA gene sequences extracted from the RAST genome annotation were analysed using the EzBioCloud (www.ezbiocloud.net) [18]. The 16S rRNA gene phylogeny was inferred with 16S rRNA gene sequences of twelve *Schaalia* type strains and *Actinomyces naeslundii* JCM 8349^T^ from the NCBI database. Similarly, multiple (*gene/loci*) sequence analyses were conducted according to previously described methods [19]. The sequences of five housekeeping genes, including *atpA (ATP synthase subunit alpha), rpoB (RNA polymerase, β-subunit), pgi (glucose-6-phosphate isomerase), metG (methionyl-tRNA synthetase), and gyrA (DNA gyrase subunit A)* were extracted from RAST genome annotation. These sequences were aligned and concatenated into a continuous sequence for a multilocus sequence analysis [20].

For core genome single-nucleotide polymorphisms (SNPs) analysis, we employed the PGAP (Prokaryotic Genome Annotation Pipeline) pangenomes analysis pipeline. This was used for the extraction and identification of SNPs from each genome sequence [21] with default parameters, except the percentage identity cutoff of the core genome SNPs was 50% and the core genome threshold was confirmed as the number of genomes employed. The core genome SNPs sequences of all genomes were compiled into a single file to construct a robust core genome SNPs phylogenetic tree.

In addition, the autoMLST (Automated Multi-Locus Species Tree) system was employed to infer the phylogeny of strains NCTC 9931 and C24, along with seven available *Schaalia* type strain genomes. *Actinomyces naeslundii* NCTC 10301^T^ was used as an outgroup. The analysis was based on core genes from genomes using the concatenated gene tree method under the default settings [22].

In the above analyses, the MEGA X (v 10.2.6) software was used to align all sequences using the MUSCLE algorithm with default settings. The maximum likelihood (ML) tree was inferred based on the Kimura two-parameter model with 1,000 bootstrap replicates. Additionally, two more 16S rRNA gene-based phylogenetic trees were built using the neighbor-joining and maximum parsimony methods in MEGA X with 1,000 bootstraps [23, 24].

### Overall genome relatedness index

The overall genome relatedness index was calculated to validate the species delineation based on average nucleotide identity (ANI), average amino acid identity (AAI), and digital DNA-DNA hybridization (dDDH) values. Genomes of strains NCTC 9931 and C24, along with available reference genomes for *Schaalia* type strains and *Schaalia odontolytica* non-type strains, as well as the outgroup *Actinomyces naeslundii* NCTC 10301^T^ and *Actinomyces israelii* DSM 43320^T^, were retrieved from the NCBI database. Digital DNA-DNA hybridization (dDDH) values were determined using the Genome-to-Genome Distance Calculator (GGDC 3.0) based on the Genome BLAST Distance Phylogeny (GBDP) *d*_*4*_ formula using the Type Stain Genome Server (TYGS) [25]. The dDDH threshold value is less than 70% for species delineation and below 79% for subspecies delineation [26, 27]. The dDDH results were visualised in a heatmap generated with the GSP 6.0 Heatmap Illustrator (Heml 2.0) tool [28].

For the ANI and AAI analyses, we computed all-vs-all distances among all uploaded genome and protein sequences (from RAST annotation). The calculation was based on both one-way ANI and AAI (best hits) and two-way ANI and AAI (reciprocal best hits) metrics using the online tool developed by Kostas lab with the recommended parameters [26, 29]. The threshold values for species delineation below 95—96% similarity for ANI and AAI, respectively. The threshold value above 96% and up to 99.5% similarity, indicates that the strains are the same species but potentially different subspecies [19, 30, 31].

### Pangenome and orthologous comparative analysis

To understand the genetic composition of species, encompassing the core, accessory, and unique genomes, two additional strains (*Schaalia odontolytica* NCTC 9935^T^ and *Schaalia meyeri* DSM 20733^T^) that were phylogenetically closest to strains NCTC 9931 and C24 were selected based on the autoMLST genome tree. We employed the Roary pipeline (Galaxy Version 3.13.0 + galaxy2) for the pangenome analysis [32]. The genomes of four strains were annotated using the Prokka Prokaryotic genome annotation tool (Galaxy Version 1.14.6 + galaxy1), keeping job resource parameters to their default settings [33, 34]. This step generated gff3 files for each genome. These gff3 files were then input into the Roary pipeline for pangenome analysis.

Within the pipeline, we set our criteria such that the minimum percentage protein sequence identity for blastp was fixed at 95%. Any gene present in at least 99% of the isolates was designated as part of the core genome, while all other parameters remained at their default settings. For visual representation of the pangenome analysis results, we utilized the BioVenn online tool [35]. For a deeper understanding of core and pan-genome functional annotations (COGs: Clusters of Orthologous Groups and KEGG: Kyoto Encyclopedia of Genes and Genomes) and comprehensive comparisons, we further employed the Bacterial Pangenome Analysis Pipeline (BPGA), known for its rapid analysis capabilities [36]. Similarly, the protein sequences of these selected strains obtained from RAST annotation were clustered based on the USEARCH Clustering Algorithm with protein sequence identity cut-off at 95%.

Furthermore, we used the Orthovenn3 web server to perform a genome comparison analysis of strains NCTC 9931 and C24 against the selected genomes of *Schaalia* type strain [37, 38]. Specifically, the protein sequences of these selected strains obtained from RAST annotation were uploaded to Orthovenn3 to perform an analysis using OrthoFinder algorithm [39] with parameter settings, inflation value at 2 and E-value at 1e-5.

### Genomic islands (GIs) prediction and pathogenicity assessment

To investigate the presence of genomic islands (GIs), *Schaalia odontolytica* NCTC 9935^T^* and Schaalia meyeri* DSM 20733^T^ were selected for comparison based on their phylogenetic proximity in the genome tree. We utilized the RAST annotated gbk files of these strains and predicted genomic islands (GIs) through the IslandViewer web server following the approaches described previously [40]. For alignment purposes, *Schaalia odontolytica XH001* served as the reference genome. We reordered the genome contigs of our strains relative to this reference using the Mauve tool [41]. The reordered genomic sequences, along with the identified GI locations, were downloaded for further visualization. To estimate bacterial pathogenicity, assembled fasta files of our strains were evaluated using the PathogenFinder 1.1 online platform with *Actinobacteria* as the organism class for a tailored analysis [42].

### Identification of virulence factor and CRISPR-Cas system analysis

The genomes of two strains were also examined alongside the type strain genomes of the *Schaalia* genus, specifically *Schaalia odontolytica* NCTC 9935^T^*and Schaalia meyeri* DSM 20733^T^. Our primary tool for virulence factor detection was the Virulence Factor Database (VFDB) [43, 44]. Utilizing this database, we aligned the nucleotide sequences of our strains against a dataset of 32,672 nucleotide sequences (retrieved on 20 August 2023) from VFDB’s comprehensive set (setB), adhering to the default parameters. We considered hits exhibiting over 70% identity as significant based on the criteria reported [45]. To delve into the CRISPR-Cas system within our strains, we employed the CRISPR-Cas +  + online tool [46]. We uploaded the genome sequences of strains NCTC 9931 and C24, *Schaalia odontolytica* NCTC 9935^T^, and *Schaalia meyeri* DSM 20733^T^ to this platform. Using the CRISPRCasFinder program, we pinpointed both the CRISPRs and the associated Cas genes within these genomes.

### Genome-based taxonomic description

To characterize the novel species, we utilized the Protologger (Galaxy Version 1.0.0) to extract additional taxonomic, functional, and ecological features [47]. The 16S rRNA gene sequences and assembled genome sequences of two novel strains were submitted to the Protologger platform via its online portal (http://www.protologger.de/). The formal description was included in compliance with the requirement of the International Code of Nomenclature of Prokaryotes (2022 Revision) [48].

## Results

### Genome profiles of novel species

The sequencing generated 1,469 Mb of raw data for both strains. After removing low-quality data, 1,183 Mb and 1,168 Mb of high-quality data were obtained for strain NCTC 9931 and C24, respectively (Table 1).

**Table 1:** Summary of sequencing data, genome assembly and annotation

Strain Name	Strain NCTC 9931	Strain C24
Raw Data (Mb)	1,469	1,469
Clean Data (Mb)	1,183	1,168
Total Reads (#)	9,796,674	9,796,674
Genome Size (bp)	2,374,847	2,345,519
Genome Completeness (%)	100%	100%
Contamination (%)	0.47%	0.47%
Genome Quality (%)	97.65%	97.65%
Contigs (#)	36	22
N50 (bp)	230,219	155,844
GC (%)	65.49	65.47
CDS (#)	2,102	2,069
RNAs (#)	52	51
Genome Coverage	498X	498X

The refined data for strain NCTC 9931, comprising 9,796,674 reads, was assembled into 36 contigs with an N50 value of 230,219 bp. This resulted in a draft genome of 2,374,847 bp with a GC content of 65.5%. A total of 9,796,674 reads for strain C24 were assembled into 22 contigs, yielding an N50 value of 155,844 bp, and the genome size of the draft genome was 2,345,519 bp with a similar GC content of 65.5%. The genome assembly quality is more than 97% for both strains, with 100% genome completeness and contamination below 0.5%, suggesting the high quality of the two assemblies (Table 1).

Subsequent RAST annotations revealed 2,102 coding sequences (CDSs) and 52 RNAs in strain NCTC 9931, while strain C24 harbored 2,069 CDSs and 51 RNAs (Table 1). Interestingly, approximately 28% of CDSs in each strain, 584 in strain NCTC 9931 and 576 in strain C24, matched with subsystem features classified into 23 distinct categories. However, a substantial proportion, over 72% of CDSs in both strains remained uncharacterized in any specific categories (Figure S1A-B).

Among the identified categories, the subsystem of protein metabolism stood out prominently in both strains, accounting for 142 CDSs in strain NCTC 9931 and 144 CDSs in strain C24. This dominance suggests that the synthesis, modification, and degradation of proteins are vital processes for these bacteria, potentially reflecting their capacity for protein metabolism and their adaptation to independent growth in the oral cavity, where proteins are often a key source of nutrients. Following protein metabolism, the subsystems of amino acids and derivatives (138 CDSs for NCTC 9931, 139 CDSs for C24), carbohydrates (109 CDSs for NCTC 9931, 106 CDSs for C24), and cofactors, vitamins, prosthetic groups, and pigments (92 CDSs for both strains) were also well-represented (Figure S1A-B). These subsystems underscore a broad metabolic repertoire, indicating that strains NCTC 9931 and C24 may possess metabolic versatility beneficial for survival in oral ecosystems.

A noteworthy observation is the presence of genes associated with virulence, disease, and defense, with strain NCTC 9931 having 22 CDSs and strain C24 possessing 21 CDSs in this category (Figure S1A-B). This hints at potential mechanisms for host interaction, suggesting that these strains might interact with hosts either as symbionts or opportunistic pathogens.

### Phylogenetic relationship analysis

**Fig. 1:**
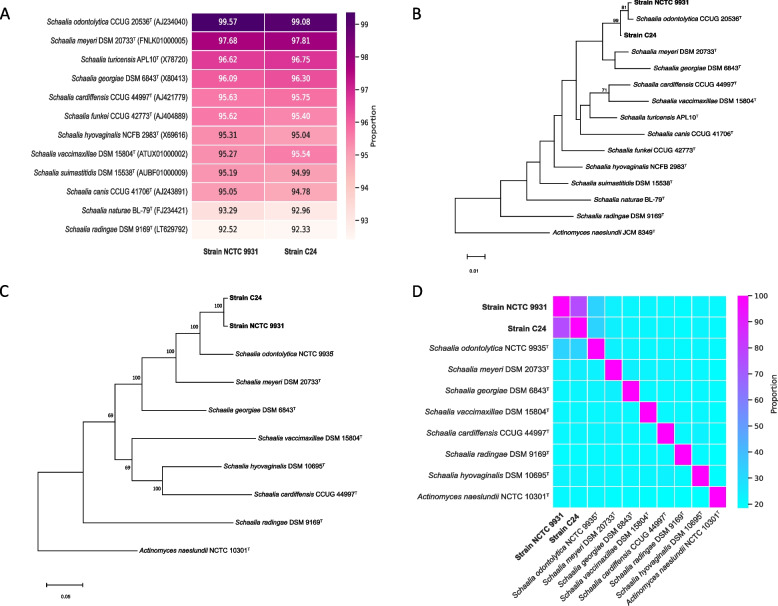
Phylogenetic and genome sequence analysis of *Schaalia* strains. **A** Nucleotide identity heatmap illustrating the similarity of 16S rRNA gene sequence of strain NCTC 9931 and strain C24 compared to *Schaalia* type strains (EzBioCloud database). Sequence similarity was calculated using the 16S-based identification tool. **B** Maximum-likelihood tree based on 16S rRNA gene sequences of both strains compared to *Schaalia* type strains. *Actinomyces neaslundii* JCM 8349^ T^ was employed as an outgroup. Bootstrap value (percentage) was computed based on 1,000 bootstrap replicates, and values with more than 50% are shown. Note that *Schaalia odontolytica* CCUG 20536^ T^ and NCTC 9935^ T^ are synonyms of the same strain. **C** autoMLST genome tree showing the relationship of strains NCTC 9931 and C24 to seven available *Schaalia* type strains. The tree was visualized using MEGA X, and bootstrap values above 50% are displayed on the tree. **D** Heatmap with dDDH values between two analysed strains and seven *Schaalia* reference type strains, the dDDH values were calculated based on the confidence interval of formula *d*_*4*_ in GBDP. *Actinomyces naeslundii* NCTC 10301^ T^ served as an outgroup. Analysed strains are highlighted in bold in this figure

The complete 16S rRNA gene sequences of strain NCTC 9931 and strain C24 were obtained from RAST genome annotation. For strain identification, these sequences underwent analysis using the 16S-based identification tool from EzBioCloud. As shown in Fig. 1A, both strains exhibited high sequence similarities to *Schaalia odontolytica* CCUG 20536^ T^ (= NCTC 9935^ T^), with identities of 99.57% and 99.08%, respectively, suggesting a close phylogenetic relationship with this species. Table 2 provides information on species and accession numbers.

**Table 2:** List of strains with nearly complete 16S rRNA gene sequences used in the phylogenetic analysis. Note that *Schaalia odontolytica* CCUG 20536^ T^ and NCTC 9935^ T^ are synonyms of the same strain

No	Strain name (T)	Accession number	Length (bp)
1	*Schaalia odontolytica* CCUG 20536^ T^	AJ234040	1,412
2	*Schaalia meyeri* DSM 20733^ T^	FNLK01000005	1,464
3	*Schaalia turicensis* APL10^T^	X78720	1,453
4	*Schaalia georgiae* DSM 6843^ T^	X80413	1,422
5	*Schaalia cardiffensis* CCUG 44997^ T^	AJ421779	1,522
6	*Schaalia funkei* CCUG 42773^ T^	AJ404889	1,395
7	*Schaalia hyovaginalis* NCFB 2983^T^	X69616	1,496
8	*Schaalia vaccimaxillae* DSM 15804^T^	ATUX01000002	1,458
9	*Schaalia suimastitidis* DSM 15538^T^	AUBF01000009	1,460
10	*Schaalia canis* CCUG 41706^T^	AJ243891	1,428
11	*Schaalia naturae* BL-79^T^	FJ234421	1,463
12	*Schaalia radingae* DSM 9169^T^	LT629792	1,473
13	Strain NCTC 9931	OQ981483	1,538
14	Strain C24	OQ981484	1,548
15	*Actinomyces naeslundii* JCM 8349^ T^	NR_113326	1,522

To further elucidate their phylogenetic positions, we downloaded the 16S rRNA gene sequences of twelve *Schaalia *type species, along with the outgroup reference type strain *Actinomyces naeslundii* JCM 8349^ T^ from the NCBI database to construct a phylogenetic tree. As shown in Fig. 1B and Figure S1C-D, strain C24 forms a distinct branch on the phylogenetic tree, highlighting potential evolutionary divergence. Whereas strain NCTC 9931, despite sharing the same branch with *Schaalia odontolytica* CCUG 20536^ T^ (= NCTC 9935^ T^), clearly shows distance based on the branch length to their common ancestor. This indicates that even in cases of high 16S rRNA sequence similarity, other aspects of genetic analysis can reveal important evolutionary differences. Further analysis is necessary to validate these findings and possibly explore additional genetic markers to clarify these phylogenetic relationships.

For further granularity, concatenated sequences of five housekeeping genes (*atpA, rpoB, pgi, metG, and gyrA*) were compared against sequences from seven available *Schaalia* type strains. The resulting maximum-likelihood tree based on these housekeeping gene sequences revealed that strains NCTC 9931 and C24 were closely related to each other but distinct from *Schaalia odontolytica* NCTC 9935^ T^ and *Schaalia meyeri* DSM 20733^ T^ (Figure S2A). While these observations provide significant insights, these phylogenetic inferences were inconclusive in determining the taxonomic positions of the strains NCTC 9931 and C24.

Core genome SNPs sequences, extracted from the genomes of strains NCTC 9931 and C24 and juxtaposed against their reference type strains, formed the foundation for another phylogenetic tree (Figure S2B). The parallels observed between the results of the core genomes SNPs analysis and the housekeeping gene analysis underpin the consistency in these analytical methodologies. However, while the core genome SNPs analysis largely corroborates the housekeeping gene results, inherent genome and gene variations across species can impact the perceived taxonomic relationships, emphasizing the importance of employing a multifaceted analysis approach for robust species classification.

### Genome relatedness analysis

Whole-genome analysis provides maximal resolution, capturing minute variations between microbes that might be overlooked in other methods [49]. Despite analyses such as 16S rRNA, housekeeping genes, and core genome SNPs, there remains a compelling need for whole-genome phylogenetic analysis to acquire the most detailed and accurate evolutionary relationships, offering robust evidence for species classification and identification. Strains NCTC 9931 and C24 form the independent branch, distinguishing them from the closely related species *Schaalia odontolytica* NCTC 9935^T^ and *Schaalia meyeri* DSM 20733^T^ (Fig. 1C and Table S1). This indicates that the strains likely represent novel species or distinct subspecies within the genus *Schaalia*. The multi-gene sequence analysis via autoMLST aligned perfectly with the core genome SNPs analysis results, reinforcing the reliability of these methodologies (Fig. 1C and Figure S2B).

The DNA-DNA hybridization (DDH) has been the gold standard in bacterial species delineation, and the digital DNA-DNA hybridization (dDDH) version offers a high-resolution tool for genome-based taxonomy, allowing for precise species demarcation in the genomic era [50–52]. To further confirm the species identities of the analysed strains, we conducted a genome comparison analysis between strains NCTC 9931 and C24 with available reference type strains using the Type (strain) Genome Server (TYGS). Neither strain NCTC 9931 nor C24 displayed dDDH values greater than 70% to any of the existing reference type strains (Fig. 1D). Interestingly, the dDDH value between these two strains stood at 75%, indicating both strains as the same species but distinct subspecies within the genus *Schaalia*.

A comparative analysis of all available *Schaalia odontolytica* genomes from the NCBI database. TYGS analysis revealed that none of the fifteen *Schaalia odontolytica* genomes (derived from three strains and twelve metagenome-assembled genomes) shared species-level identity with strains NCTC 9931 and C24 (referenced in Figure S2C and Table S2). However, *Schaalia odontolytica* ATCC 17982 showed a 100% dDDH value with strain NCTC 9931, indicating that they are the same species (Figure S2D), Based on the records of culture collections, strain ATCC 17982 was a subculture of strain NCTC 9931 (10.13145/bacdive156.20230509.8.1). When both strains ATCC 17982 and NCTC 9931 were compared to *Schaalia odontolytica* NCTC 9935^ T^, their phylogenetic relationship was distant, with dDDH values below 70%, suggesting that they should not have been classified as *Schaalia odontolytica*. These observations also suggest that the strains displaying the same species identity to strain NCTC 9931 should be reclassified, to properly reflect their taxonomic status.

**Fig. 2:**
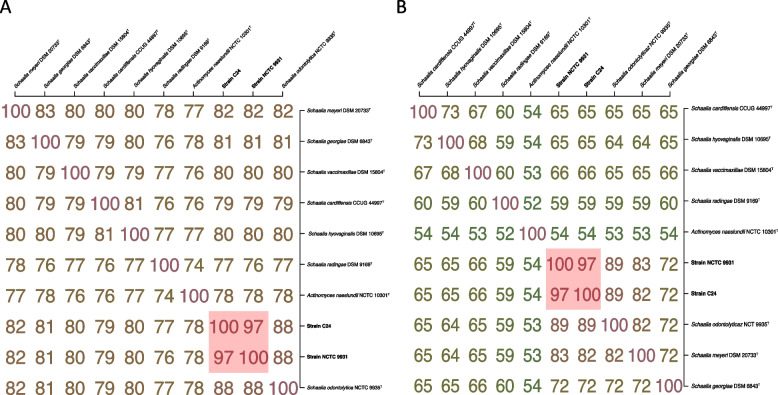
ANI and AAI analysis. **A** Matrix with ANI results between two analysed strains and seven *Schaalia* reference type strains, ANI values were estimated using both best hits (one-way ANI) and reciprocal best hits (two-way ANI) between two genomic datasets. **B** Matrix with AAI results between two analyzed strains and seven *Schaalia* reference type strains, AAI values were estimated using both best hits (one-way AAI) and reciprocal best hits (two-way AAI) between two genomic datasets. *Actinomyces naeslundii* NCTC 10301^T^ was employed as an outgroup species. The pink-shaded area indicates the values with ANI and AAI > 96%, and the analyzed strains are highlighted in bold in the matrix

Subsequent ANI analyses revealed that neither strains NCTC 9931 nor C24 exhibited ANI values greater than 95% when compared to other available reference type strains. The ANI value between the two strains is 97% (Fig. 2A). This suggests that strains NCTC 9931 and C24 represent novel species within the genus *Schaalia* and can be further differentiates as distinct subspecies. Additional ANI analysis also showed that strain ATCC 17982 shares significant genomic similarity with strain NCTC 9931 and C24, with ANI values of 100% and 97%, respectively (Figure S3). The 100% ANI value between strains ATCC 17982 and NCTC 9931 confirmed the clonality of both strains [53]*.* In addition, AAI analysis of strains NCTC 9931 and C24 revealed consistent results with ANI analysis (Fig. 2B), further supporting their distinct taxonomic positions within the genus *Schaalia*.

### Pangenome analysis elucidates the genetic landscape and diversification

**Fig. 3:**
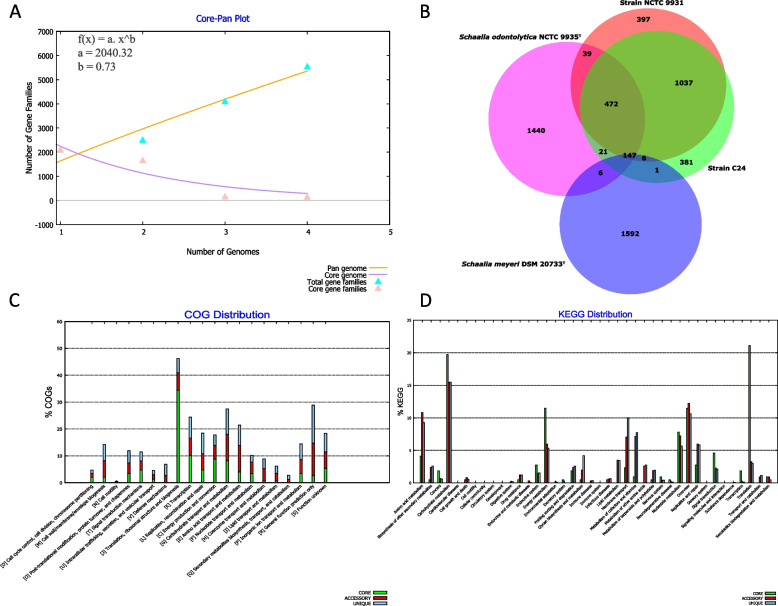
Pangenome analysis by BPGA. **A** The plot of the number of gene families in the core and pangenome against the number of genomes of four selected strains. **B** Venn diagram showing the numbers of core, accessory, and unique genes present in four selected strains. **C** COGs distribution of core, accessory, and unique genes of four selected strains. The distribution highlights the functional roles of genes within each category. **D** Distribution of KEGG pathway classification in core, accessory, and unique genomes in the four strains. The four selected strains are NCTC 9931, C24, *Schaalia odontolytica* NCTC 9935^T^, and *Schaalia meyeri* DSM 20733^T^

To comprehensively understand the genetic composition of novel species, encompassing the core, accessory, and unique genomes, we selected *Schaalia odontolytica* NCTC 9935^T^ and *Schaalia meyeri* DSM 20733^T^ that were phylogenetically closest to strains NCTC 9931 and C24 based on the genome tree to perform the pangenome analysis with a blastp sequence identity cut-off of 95% using BPGA and Roary [32, 36, 54]. The core-pan genome plot showcased the varying number of gene families as the analysed genomes increased (Fig. 3A). The core genome curve indicates the consistent gene families across all strains, while the pangenome curve represents the cumulative gene families. Mathematical modeling revealed a power-law relationship for the pangenome, given by f(x) = a.x^b with parameters a = 2,040.32 and b = 0.73 (Fig. 3A). The open nature of the pangenome, implies ongoing genetic diversification within the *Schaalia* genus, emphasizing the potential for discovering new genes as more genomes are sequenced.

A marked genetic distinctiveness was observed when the BPGA pangenome analysis was conducted using a stringent 95% protein sequence identity cut-off: a core of 147 gene families was conserved across the four strains. This contrasted sharply with the unique genetic footprints of the two novel species and the referenced strains (Fig. 3B). Roary's computation of the pangenome and core genome sizes (5,271 and 165, respectively) resonated well with BPGA's findings (Figure S4). Notably, a side-by-side comparison of strains NCTC 9931 and C24 revealed a shared genetic backbone of 1,662 core gene families. However, strain-specific signatures emerged with NCTC 9931 and C24 boasting 436 and 403 unique gene families, respectively (Fig. 3B). This genetic demarcation not only accentuates their distinct biological functionalities but also fortifies their status as unique subspecies.

Delving deeper into the functional landscape of the core, accessory, and unique gene families, we embarked on the COGs and KEGG functional analysis for the quartet of strains. The COGs distribution spotlighted four predominant categories: information storage and processing, metabolism, cellular processes and signaling, and a cohort of poorly characterized genes (Figure S5A). The core genes primarily steered translation, ribosomal structure and biogenesis, transcription, energy production, and carbohydrate metabolism (Fig. 3C). In contrast, accessory and unique genes had a predilection for general function prediction, carbohydrate transport, and amino acid metabolism.

The KEGG distribution, on the other hand, painted a vivid picture of six major categories, with a heavy inclination of core genes towards translation, carbohydrate metabolism, and energy metabolism (Fig. 3D & S5B). The accessory genes showcased a diverse array, from carbohydrate metabolism to amino acid metabolism and membrane transport. Collectively, these findings weave a tale of both shared heritage and individual genetic evolution, underscoring the complexity and diversity harboured within the *Schaalia* genus.

### Functional comparative analysis

**Fig. 4:**
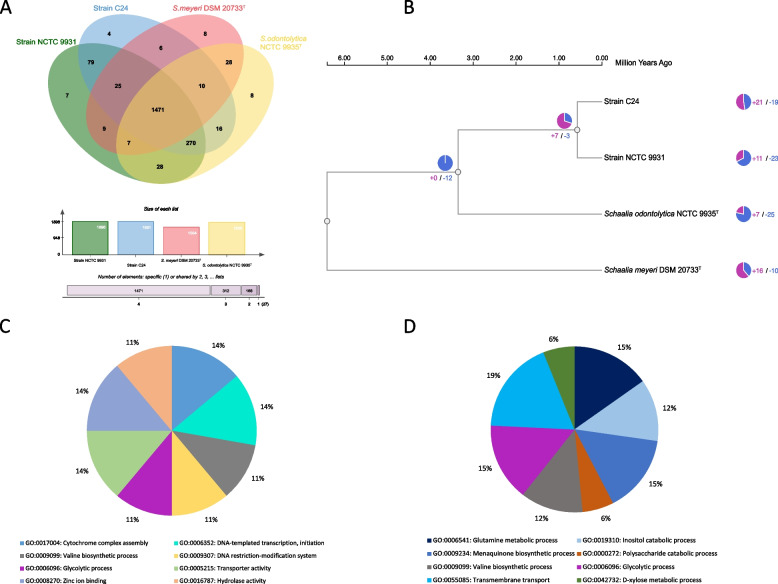
Orthologous cluster identification and comparative analysis. **A** Representation of orthologous gene clusters across four selected strains. The Venn diagram illustrates the unique and shared orthologous gene clusters, complemented by a bar chart that quantitatively details the number of clusters for each strain. **B** Analysis of gene family evolution in the four strains, highlighting contracted and expanded gene families. The pie chart visually contrasts the number of contracted (in purple) versus expanded (in blue) gene families, displaying the evolution of gene families and differences between species. **C** GO enrichment analysis for 21 expanded gene families matched eight GO categories in strain C24. **D** GO enrichment analysis for 11 expanded gene families matched eight GO categories in strain NCTC 9931. The four strains analysed are NCTC 9931, C24, *Schaalia odontolytica* NCTC 9935^T^, and *Schaalia meyeri* DSM 20733^T^

To gain insights into the genetic composition and potential functional differences among our strains of interest, we utilized the OrthoVenn3 web server for whole-genome comparisons; we discerned that the quartet collectively harboured 1,471 shared orthologous gene clusters (Fig. 4A). Specifically, strain NCTC 9931 demonstrated overlaps of 1,845, 1,512, and 1,776 orthologous gene clusters with strain C24, *Schaalia meyeri* DSM 20733^T^, and *Schaalia odontolytica* NCTC 9935^T^, respectively. Singletons were found to be most abundant in *Schaalia odontolytica* NCTC 9935^T^ [155], followed by strain NCTC 9931 [86], *Schaalia meyeri* DSM 20733^T^ [106], and strain C24 [67] (Table S3). When juxtaposed, strains NCTC 9931 and C24 exhibited 51 and 36 exclusive unique gene clusters, respectively, underscoring their evolutionary and functional disparities.

Delving deeper, gene family expansions and contractions elucidated evolutionary trajectories. Relative to their common ancestor, strain NCTC 9931 had 11 expanded and 23 contracted gene families, while strain C24 manifested expansions in 21 and contractions in 19 gene families. This substantiates their genetic divergence not only from each other but also from *Schaalia meyeri* DSM 20733^ T^ and *Schaalia odontolytica* NCTC 9935^ T^ (Fig. 4B).

Gene Ontology (GO) enrichment analysis of the expanded gene families in strain C24 spotlighted eight principal categories, including cytochrome complex assembly, DNA-templated transcription initiation, and glycolytic processes, among others (Fig. 4C & Figure S6A). Strain NCTC 9931's expanded gene set, on the other hand, was associated with processes like glutamine metabolism, inositol catabolism, and transmembrane transport (Fig. 4D & Figure S6B). The contracted gene families in strain C24 were involved in 22 biological processes and two cellular components, while those in NCTC 9931 were mapped to 12 biological processes and a singular molecular function (Figure S6C & S6D).

These differential GO enrichments fortify the taxonomical distinctions between strain NCTC 9931 and strain C24. They also suggest distinct adaptive and evolutionary pathways, perhaps indicative of varied ecological niches or functional roles these strains might play in their respective environments.

### Genomic features and characterisation

To gain deeper insights into the properties and functions of two novel species, we conducted genomic island (GIs) analysis by comparing them to the closest two reference strains *Schaalia odontolytica* NCTC 9935^T^ and *Schaalia meyeri* DSM 20733^T^. Our analysis revealed that strains NCTC 9931 and C24 harbored 15 and 13 putative GIs, respectively, while *Schaalia odontolytica* NCTC 9935^T^ has 20 putative GIs and *Schaalia meyeri* DSM 20733^T^ possess 12 GIs (Figure S7). Among the 15 GIs identified in the assembled genome of strain NCTC 9931, the largest GI was 35,492 bp, containing 30 genes, while the second largest GI has a size of 25,997 bp, consisting of 23 genes (Table S4). For strain C24, the largest GIs were 21,984 bp, with 20 genes. In contrast, the top GIs of *Schaalia odontolytica* NCTC 9935^T^ were 28,369 bp and 27,615 bp, encompassing 24 and 33 genes, individually (Table S4). The top GIs of *Schaalia meyeri* DSM 20733^T^ were 19,739 bp with 22 genes. GIs are gene clusters predicted to have integrated into the genome through horizontal transfer and often contain functions related to adaptation to the environment [19]. These clear differences in GIs between strains NCTC 9931 and C24 compared to the reference *Schaalia odontolytica* NCTC 9935^T^ and *Schaalia meyeri* DSM 20733^T^ provide further evidence for classification as novel species.

Pathogenicity analysis of strains NCTC 9931 and C24 revealed their potential human pathogenicity risks by matching to the same organism *Bifidobacterium dentium* of the class *Actinomycetes* (Table S5). By comparing the genomes of the four *Schaalia strains* (NCTC 9931, C24, *Schaalia odontolytica* NCTC 9935^ T^, and *Schaalia meyeri* DSM 20733^ T^) against 32,672 virulence factors in the VFDB database, we found that 14 virulence-associated factors in strain NCTC 9931, these factors mainly including stress survival (*sodA*), nutritional/metabolic factor (*glnA1, leuD, narH, narG, narX, ctpC, sugC*), regulation (*relA, sigA/rpoV*), immune modulation (*nuoG, rmlA*) and adherence (*groEL2, AvisC_010100012015*). In strain C24, a total of 11 genes encoding virulence-associated factors were identified*,* including stress survival (*sodA*), nutritional/metabolic factor (*leuD, ctpC, narH, glnA1, sugC*), regulation (*relA, sigA/rpoV*), immune modulation (*nuoG, rmlA*) and adherence (*groEL2*) (Table 3).

**Table 3:** Virulence factors were predicted in four selected strains against the VFDB database

Strain	VF ID	Gene Name	Gene Product	Size (bp)	Nucleotide Identity (%)	Evalue	Function
NCTC 9931	VF0816	*glnA1*	type I glutamate–ammonia ligase	1211	75	4.22E-135	Nutritional/Metabolic factor
NCTC 9931	VF0287	*relA*	Probable GTP pyrophosphokinase RelA	474	73	1.05E-31	Regulation
NCTC 9931	VF0257	*sigA/rpoV*	Probable RNA polymerase sigma factor RpoD	714	82	1.39E-173	Regulation
NCTC 9931	VF0814	*leuD*	3-isopropylmalate dehydratase small subunit	261	83	1.63E-58	Nutritional/Metabolic factor
NCTC 9931	VF0866	*groEL2*	molecular chaperone GroE	1553	79	0	Adherence
NCTC 9931	VF0302	*narH*	nitrate reductase subunit beta	1387	76	0	Nutritional/Metabolic factor
NCTC 9931	VF0302	*narG*	nitrate reductase subunit alpha	870	77	4.46E-132	Nutritional/Metabolic factor
NCTC 9931	VF0304	*sodA*	Probable superoxide dismutase (Mn)	552	74	5.00E-52	Stress survival
NCTC 9931	VF0820	*narX*	nitrate reductase	210	77	2.41E-25	Nutritional/Metabolic factor
NCTC 9931	VF0848	*ctpC*	manganese-exporting P-type ATPase CtpC	107	82	8.10E-15	Nutritional/Metabolic factor
NCTC 9931	VF0833	*nuoG*	NADH-quinone oxidoreductase subunit G	203	80	4.13E-35	Immune modulation
NCTC 9931	VF0841	*rmlA*	glucose-1-phosphate thymidylyltransferase RfbA	847	76	1.28E-109	Immune modulation
NCTC 9931	VF0842	*sugC*	sn-glycerol-3-phosphate ABC transporter ATP-binding protein UgpC	40	93	2.28E-06	Nutritional/Metabolic factor
NCTC 9931	VF1201	*AvisC_010100012015*	surface-anchored fimbrial subunit	421	75	2.91E-42	Adherence
Strain C24	VF0257	*sigA/rpoV*	Probable RNA polymerase sigma factor RpoD	714	82	2.40E-173	Regulation
Strain C24	VF0814	*leuD*	3-isopropylmalate dehydratase small subunit	261	83	2.83E-58	Nutritional/Metabolic factor
Strain C24	VF0848	ctpC*ctpC*	manganese-exporting P-type ATPase CtpC	107	82	7.92E-15	Nutritional/Metabolic factor
Strain C24	VF0302	*narH*	nitrate reductase subunit beta	1387	76	0	Nutritional/Metabolic factor
Strain C24	VF0304	*sodA*	Probable superoxide dismutase (Mn)	552	74	4.44E-52	Stress survival
Strain C24	VF0841	*rmlA*	glucose-1-phosphate thymidylyltransferase RfbA	847	76	4.76E-106	Immune modulation
Strain C24	VF0816	*glnA1*	type I glutamate–ammonia ligase	1211	74	1.53E-130	Nutritional/Metabolic factor
Strain C24	VF0287	*relA*	Probable GTP pyrophosphokinase RelA	474	73	6.57E-34	Regulation
Strain C24	VF0842	*sugC*	sn-glycerol-3-phosphate ABC transporter ATP-binding protein UgpC	40	93	3.84E-06	Nutritional/Metabolic factor
Strain C24	VF0833	*nuoG*	NADH-quinone oxidoreductase subunit G	203	80	1.91E-33	Immune modulation
Strain C24	VF0866	*groEL2*	molecular chaperone GroE	1553	79	0	Adherence
*Schaalia odontolytica* NCTC 9935^ T^	VF0287	*relA*	Probable GTP pyrophosphokinase RelA	1382	70	1.18E-40	Regulation
*Schaalia odontolytica* NCTC 9935^ T^	VF0866	*groEL2*	molecular chaperone GroE	1549	78	0	Adherence
*Schaalia odontolytica* NCTC 9935^ T^	VF0847	*mpa*	proteasome ATPase	315	75	2.38E-32	Stress survival
*Schaalia odontolytica* NCTC 9935^ T^	VF0848	*ctpC*	manganese-exporting P-type ATPase CtpC	107	82	2.20E-14	Nutritional/Metabolic factor
*Schaalia odontolytica* NCTC 9935^ T^	VF0841	*rmlA*	glucose-1-phosphate thymidylyltransferase RfbA	847	75	9.45E-101	Immune modulation
*Schaalia odontolytica* NCTC 9935^ T^	VF0304	*sodA*	Probable superoxide dismutase (Mn)	552	73	1.64E-49	Stress survival
*Schaalia odontolytica* NCTC 9935^ T^	VF0257	*sigA/rpoV*	Probable RNA polymerase sigma factor RpoD	716	81	2.22E-154	Regulation
*Schaalia odontolytica* NCTC 9935^ T^	VF0833	*nuoG*	NADH-quinone oxidoreductase subunit G	142	82	5.71E-25	Immune modulation
*Schaalia odontolytica* NCTC 9935^ T^	VF0846	*zmp1*	M13 family metallopeptidase	444	79	5.99E-80	Exoenzyme
*Schaalia odontolytica* NCTC 9935^ T^	VF0302	*narH*	nitrate reductase subunit beta	1387	75	7.32E-180	Nutritional/Metabolic factor
*Schaalia odontolytica* NCTC 9935^ T^	VF0302	*narG*	NADH-quinone oxidoreductase subunit G	870	76	1.32E-122	Nutritional/Metabolic factor
*Schaalia odontolytica* NCTC 9935^ T^	VF0820	*narX*	nitrate reductase	210	77	1.53E-22	Nutritional/Metabolic factor
*Schaalia meyeri* DSM 20733^ T^	VF0841	*rmlA*	glucose-1-phosphate thymidylyltransferase RfbA	766	74	1.06E-82	Immune modulation
*Schaalia meyeri* DSM 20733^ T^	VF0842	*groEL2*	molecular chaperone GroE	1553	78	0	Adherence
*Schaalia meyeri* DSM 20733^ T^	VF0842	*sugC*	sn-glycerol-3-phosphate ABC transporter ATP-binding protein UgpC	638	75	1.66E-64	Nutritional/Metabolic factor
*Schaalia meyeri* DSM 20733^ T^	VF0300	*ideR*	Iron-dependent repressor and activator IdeR	357	74	4.86E-30	Regulation
*Schaalia meyeri* DSM 20733^ T^	VF0304	*sodA*	Probable superoxide dismutase (Mn)	552	73	2.77E-40	Stress survival
*Schaalia meyeri* DSM 20733^ T^	VF0833	*nuoG*	NADH-quinone oxidoreductase subunit G	262	77	1.68E-32	Immune modulation
*Schaalia meyeri* DSM 20733^ T^	VF0815	*lysA*	diaminopimelate decarboxylase	134	80	1.51E-16	Nutritional/Metabolic factor

All four strains share key genes associated with stress survival (*sodA*), regulation (*relA, sigA/rpoV*), and immune modulation (*nuoG, rmlA*), showcasing common pathogenic mechanisms. Notably, the absence of the exoenzyme-associated gene *zmp1* (encoding putative zinc-dependent metalloprotease-1) in strain NCTC 9931, C24, and *Schaalia meyeri* DSM 20733^ T^ compared to *Schaalia odontolytica* NCTC 9935^ T^ implies variations in their interaction with the host, which could impact their infection mechanisms and pathogenicity, and may lead to different pathological processes and clinical manifestations. Additionally, while most strains share common metabolic genes, their composition and number vary (Table 3 and Figure S8), indicating potential differences in nutritional strategies and environmental adaptation. A Venn diagram (Figure S8) visually summarizes these findings, showing the unique and shared virulence factors across the strains.

CRISPR-Cas analysis for novel species identified 9 CRISPRs and 14 Cas genes in strain NCTC 9931, with the largest CRISPR array consisting of 122 spacers (Table S6 & Table S7). In contrast, stain C24 exhibited only five CRISPRs without Cas, and the largest CRISPR array consisted of only four spacers (Table S6 & Table S7). *Schaalia odontolytica* NCTC 9935^ T^ possessed ten CRISPRs and one Cas gene, while *Schaalia meyeri* DSM 20733^ T^ also has nine CRISPRs without the Cas gene. Notably, strain NCTC 9931 hosted a greater number of Cas types and subtypes (Table S7), suggesting that strain NCTC 9931 may have significant functional defenses compared to the other three strains. The CRISPR-Cas system serves as an adaptive immune system found in bacteria, providing protection against foreign genetic elements such as bacteriophages and plasmids. CRISPR-Cas analysis suggests that strain NCTC 9931 might have encountered a wide variety of genetic invaders in its environment and evolved a robust defence mechanism against them. A higher number of CRISPR-Cas genes signifies a greater potential for adaptability and survival in challenging environments.

## Discussion

In this study, the genomic landscapes of strains NCTC 9931 and C24, as unveiled by whole-genome sequencing, offer pivotal insights into their biology and evolutionary history. The sequencing quality and subsequent annotations affirm the robustness of the assembled genomes. Both strains, despite their close phylogenetic proximity to *Schaalia odontolytica*, distinguish themselves through various analyses, cementing their status as novel taxa within the genus *Schaalia*.

While phylogenetic analyses based on 16S rRNA, housekeeping genes, and core genome SNPs can suggest genetic relatedness, they often fall short in definitively characterizing organisms as novel species or subspecies [56, 57]. A comprehensive genome dissection and comparative analysis provide a more substantial foundation for species identification [52]. In this context, dDDH, ANI, and AAI emerge as pivotal metrics. These methodologies fundamentally rely on whole-genome comparisons rather than individual or a handful of genes, making them the gold standard for bacterial species identification, widely accepted across the scientific community [58–60]. Interestingly, while ANI and AAI thresholds for subspecies demarcation remain debatable, our analysis of strains NCTC 9931 and C24 revealed 97% ANI, suggesting a notable divergence. We argue that this 3% difference could signify subspecies-level variations. Consequently, in our study, values below 99.5% for ANI and AAI were designated as indicative of novel subspecies. This threshold, supported by our comprehensive pangenome and functional characterization, highlights the genomic distinctions between the two strains. The threshold range for subspecies boundary was also supported by previous study cases [19, 30, 31, 61].

It is critical to note that the choice of reference genomes is vital for the classification and nomenclature of new species. Consequent to the International Code of Nomenclature of Prokaryotes (ICNP) and the current standard for the use of genome data for the taxonomy of prokaryotes [53, 62, 63], analyses for new taxa should include genomes of effectively published type strains with validly published name. Non-type strain genomes may serve only as comparative references and should not be used as references for taxa delineation. In our analysis of the genus *Schaalia*, fewer than the twelve type strains of *Schaalia* species with validly published name had available genomes (https://lpsn.dsmz.de/genus/schaalia). None of the analysed genomes of strains NCTC 9931 and C24 showed high similarity to any existing type strain genomes. However, when extended comparisons were made with non-type *Schaalia odontolytica*, several genomes exhibited high similarity (dDDH value > 70% and ANI > 95%) to our strains NCTC 9931 and C24, particularly *Schaalia odontolytica* ATCC 17982, which showed a 100% similarity to NCTC 9931 (Figure S2C-D & S3). That is because strain ATCC 17982 was passaged from strain NCTC 9931 (https://www.atcc.org/products/17982#detailed-product-information).

For pangenome analysis, the choice of reference organisms is crucial. In our study, the genomes of *Schaalia odontolytica* NCTC 9935^T^ and *Schaalia meyeri* DSM 20733^T^, which are evolutionarily closest to strains NCTC 9931 and C24, were selected. Significant disparities between our strains and their closest relatives strengthen their identity as distinct species. However, a consensus on the protein sequence identity threshold for pangenome analysis remains elusive. While many studies default to a 50% blastp threshold [64–67], our analyses (both BPGA and Roary) employed a stringent 95% blastp threshold [54, 68], which ensures that only sequences with very high similarity are deemed homologous. Given the close evolutionary ties and subtle differences expected between NCTC 9931 and C24, a higher threshold may help in discerning highly conserved core genes from accessory and unique genes, minimizing false positives [68, 69].

In our orthologous gene cluster comparison using OrthoVenn3, an inflation value of 2 was adopted, yielding consistent results with other tested values (1.5 and 5). It's important to note that the algorithmic differences between the Markov Clustering Algorithm (MCL) in OrthoVenn3 and USEARCH in pangenome tools can lead to varying gene cluster definitions [21, 32, 70]. Therefore, the core genome cluster obtained in pangenome analysis was 147, while the shared orthologous gene clusters were 1471. Despite these discrepancies, the overarching findings from both analyses converged, highlighting the strains' unique genomic signatures. The expansion and contraction of gene families in strains NCTC 9931 and C24 hint at the evolutionary pressures they've faced, such as gene duplication, loss, or horizontal gene transfer. These shifts can influence their survival strategies and interactions in their environments [71].

Our study also unveiled horizontally transferred Genomic Islands (GIs) that often harbor genes essential for survival in specific environments, conferring advantages such as antibiotic resistance or virulence [72, 73]. Although we have no direct evidence pointing to the pathogenic potential of strains NCTC 9931 and C24, our prediction analysis indicates their potential pathogenic risk. Genes like *sodA*, crucial for microbial oxidative stress resistance, and *nuoG* and *rmlA*, important for immune modulation, were identified. The *sodA* gene encodes for Mn-SOD in bacteria, which has a critical antioxidative function. Mn-SOD aids bacteria in defending against assaults from the host immune system, particularly from superoxide anions produced by neutrophils. This capability allows bacteria to survive and multiply within the host, thereby amplifying their pathogenicity [74, 75]. The *rmlA* gene is responsible for producing the enzyme glucose-1-phosphate thymidylyltransferase. This enzyme plays a crucial role in the biosynthesis pathways of bacterial lipopolysaccharide (LPS) and other surface polysaccharides, and has been found to have a direct impact on the pathogenicity of bacteria [76, 77]. The presence of these genes underscores the need for careful research into these strains and vigilance regarding their pathogenic potential in oral diseases.

Additionally, the identification of the significant number of CRISPR-Cas elements in strain NCTC 9931 offers insights into its adaptive immune mechanisms against foreign genetic elements. In contrast, the limited system in C24 might indicate its reliance on alternate protective measures or a more stable and shielded environment. The CRISPR-Cas system serves as an adaptive immune mechanism in bacteria and archaea, offering protection against foreign genetic entities such as bacteriophages and plasmids [78, 79]. Central to the Type I CRISPR-Cas system is the Cas3 protein, which is responsible for the cleavage of target DNA [80]. Besides, the Cas1 protein, in tandem with Cas2, plays a pivotal role in spacer acquisition [81]. These spacers act as historical records, offering insights into previous encounters with phages and plasmids [82]. Given this information, it can be inferred that the NCTC 9931 bacterium, equipped with the CRISPR-Cas system—notably the Type I and Type I-E variants, possesses a sophisticated adaptive immunity mechanism, enabling it to ward off intruding genetic material.

In the broader perspective, understanding *Schaalia*'s interactions in the oral microbiome, its adaptive responses, and its implications in systemic diseases becomes vital in the current research landscape. As antibiotic resistance surges, delving deep into genera like *Schaalia* becomes increasingly urgent. The genomic blueprints of *Schaalia* species are repositories of evolutionary tales, metabolic pathways, and potential virulence mechanisms. Tools like dDDH and ANI & AAI have demystified their evolutionary connections and have distinguished them from other genera. As we usher into an advanced phase of microbial research, *Schaalia* stands as a beacon, emphasizing that every sequenced genome and studied interaction enriches our grasp on the intricate tapestry of life.

## Conclusions

Whole-genome sequence analyses indicated the phylogenetic placement of strains, NCTC 9931 and C24, as independent lineages. Furthermore, overall genome-relatedness index analyses confirmed the taxonomic positions of these strains as belonging to the same species but distinct subspecies within the genus *Schaalia*. The taxonomic placements of the strains were also supported by the pangenome and orthologous gene cluster analyses which also highlighted their differences in gene properties and biological functions. The expansion and contraction of gene families in both strains hint at their evolutionary pressures and functional differences. Furthermore, the presence of CRISPR and Cas in strain NCTC 9931 genes indicated a robust defense mechanism. By providing in-depth genomic analyses on two novel strains, this study lays foundational groundwork for future studies, paving the way for novel insights and breakthroughs in oral bacteria research.

### Description of *Schaalia dentiphila* sp. nov.

*Schaalia dentiphila* (den.ti’phi.la. L. masc. n. *dentes*, teeth; N.L. fem. adj. suff. *-phila*, friend, loving; from Gr. fem. adj. philê, loving; N.L. fem. adj. *dentiphila*, teeth-loving).

Cells are Gram-stain positive rods. Smooth colonies produce haemolysis on Columbia blood agar. Utilise fructose but not L-arabinose, inositol, mannitol, raffinose and trehalose as sole carbohydrate source for growth. Genome prediction shows starch utilization while sulfide and L-serine utilization for L-cysteine and acetate production (EC:2.3.1.30, 2.5.1.47). Additional phenotypic characteristics as summarized in Table 4.

**Table 4:** Phenotypic characteristics of strains NCTC 9931 and C24, phenotypic characteristics extracted from BacDive [55] and previous report [8]

Characters	Strain NCTC 9931	Strain C24
Cell morphology	Rods	Rods, with frequent clubbing
Cell length (µm)	4	3
Colony morphology on Columbia blood agar	Smooth brownish colonies with 0.5–1.0 mm in diameter with α-haemolysis	Smooth red colonies with 0.5–1.0 mm in diameter with β-haemolysis
**Sole carbohydrate utilization**		
L-Arabinose	-	-
Mannitol	-	-
Mannose	+	-
Raffinose	-	-
Trehalose	-	-
Nitrate reduction	+	-
Fructose	+	+
Inositol	-	-

The type strain NCTC 9931^T^ (= ATCC 17982^T^ = DSM 43331^T^ = CIP 104728^T^ = CCUG 18309^T^ = NCTC 14978^T^ = CGMCC 1.90328^T^) was isolated from carious lesions of the human dentine and has a genome size of 2.37 Mbp with a GC content of 65.5%.

### Description of *Schaalia dentiphila* subsp. *dentiphila* subsp. nov.

*Schaalia dentiphila* subsp. *dentiphila* (den.ti’phi.la. L. masc. n. *dentes*, teeth; N.L. fem. adj. suff. *-phila*, friend, loving; from Gr. fem. adj. philê, loving; N.L. fem. adj. *dentiphila*, teeth-loving).

Utilise mannose as sole carbohydrate source for growth. Reduce nitrate. Smooth brownish colonies with 0.5–1.0 mm in diameter with α-haemolysis on Columbia blood agar. Additional phenotypic characteristics as summarized in Table 4.

The type strain NCTC 9931^T^ (= ATCC 17982^T^ = DSM 43331^T^ = CIP 104728^T^ = CCUG 18309^T^ = NCTC 14978^T^ = CGMCC 1.90328^T^) has a genome size of 2.37 Mb with a GC content of 65.5%.

### Description of *Schaalia dentiphila* subsp. *denticola* subsp. nov.

*Schaalia dentiphila* subsp. *denticola* (den.ti.co.la. L. masc. n. *dens* (gen. *dentis*), tooth; L. masc./fem. n. suff. -*cola*, inhabitant, dweller; from L. masc./fem. n. *incola*, dweller; N.L. masc./fem. n. *denticola*, tooth dweller).

Smooth red colonies with 0.5–1.0 mm in diameter with β-haemolysis on Columbia blood agar. Additional phenotypic characteristics as summarized in Table 4.

The type strain C24^T^ (= NCTC 14980^T^ = CGMCC 1.90328^T^) was isolated from supragingival plaque-associated root surface caries in Papua New Guinea indigenes and has a genome size of 2.35 Mb with a GC content of 65.5%.

### Supplementary Information

Supplementary Material 1.

## Data Availability

The genome sequence and 16S rRNA gene sequence of strain NCTC 9931 and C24 were deposited into the GenBank database. The accession number for 16S rRNA gene sequences for strains NCTC 9931 and C24 are OQ981483 and OQ981484, respectively. The accession number of whole genome sequences for strain NCTC 9931 and C24 were assigned as JASPFE000000000 and JASPEY000000000, respectively. These genome sequences can be accessed by searching PRJNA976213 in the NCBI database (https://www.ncbi.nlm.nih.gov/). The accession number of other genome sequences used in this study can be found in Supplementary Table S1-S2. The strains NCTC 9931 and C24 used in this study are available from the collections of the National Collection of Type Cultures (NCTC), United Kingdom, and China General Microbiological Culture Collection Center (CGMCC), China.

## References

[1] Nouioui I, Carro L, Garcia-Lopez M, Meier-Kolthoff JP, Woyke T, Kyrpides NC (2018). Genome-based taxonomic classification of the phylum actinobacteria. Front Microbiol.

[2] Villmones HC, Svanevik M, Ulvestad E, Stenstad T, Anthonisen IL, Nygaard RM (2022). Investigating the human jejunal microbiota. Sci Rep.

[3] Herreros-Pomares A, Hervas D, Bagan-Debon L, Jantus-Lewintre E, Gimeno-Cardona C, Bagan J (2023). On the oral microbiome of oral potentially malignant and malignant disorders: dysbiosis, loss of diversity, and pathogens enrichment. Int J Mol Sci.

[4] Cronin JT, Richards BW, Skedros JG (2023). Schaalia (Formerly Actinomyces) turicensis infection following open rotator cuff repair. Cureus.

[5] Staskova A, Sondorova M, Nemcova R, Kacirova J, Madar M (2021). Antimicrobial and antibiofilm activity of the probiotic strain streptococcus salivarius K12 against oral potential pathogens. Antibiotics (Basel).

[6] Parte AC, Sarda Carbasse J, Meier-Kolthoff JP, Reimer LC, Goker M (2020). List of prokaryotic names with standing in nomenclature (LPSN) moves to the DSMZ. Int J Syst Evol Microbiol.

[7] Sayers EW, Bolton EE, Brister JR, Canese K, Chan J, Comeau DC (2023). Database resources of the National Center for Biotechnology Information in 2023. Nucleic Acids Res..

[8] Loo CY. Surface properties and colonization potential of actinomyces [D]: University of Sydney; 1994. https://ses.library.usyd.edu.au/handle/2123/4709.

[9] Hill PE, Knox KW, Schamschula RG, Tabua J (1977). The identification and enumeration of actinomyces from plaque of New Guinea indigenes. Caries Res..

[10] Schmieder R, Edwards R (2011). Quality control and preprocessing of metagenomic datasets. Bioinformatics.

[11] Petit RA, Read TD (2020). Bactopia: a Flexible Pipeline for Complete Analysis of Bacterial Genomes. mSystems.

[12] Gurevich A, Saveliev V, Vyahhi N, Tesler G (2013). QUAST: quality assessment tool for genome assemblies. Bioinformatics..

[13] Parks DH, Imelfort M, Skennerton CT, Hugenholtz P, Tyson GW (2015). CheckM: assessing the quality of microbial genomes recovered from isolates, single cells, and metagenomes. Genome Res.

[14] Nishimura O, Hara Y, Kuraku S (2017). gVolante for standardizing completeness assessment of genome and transcriptome assemblies. Bioinformatics.

[15] Manni M, Berkeley MR, Seppey M, Zdobnov EM. BUSCO: Assessing Genomic Data Quality and Beyond. Curr Protoc. 2021;1(12). 10.1002/cpz1.323 34936221

[16] Brettin T, Davis JJ, Disz T, Edwards RA, Gerdes S, Olsen GJ (2015). RASTtk: a modular and extensible implementation of the RAST algorithm for building custom annotation pipelines and annotating batches of genomes. Sci Rep..

[17] Pruitt KD, Tatusova T, Maglott DR (2007). NCBI reference sequences (RefSeq): a curated non-redundant sequence database of genomes, transcripts and proteins. Nucleic Acids Res.

[18] Yoon SH, Ha SM, Kwon S, Lim J, Kim Y, Seo H (2017). Introducing EzBioCloud: a taxonomically united database of 16S rRNA gene sequences and whole-genome assemblies. Int J Syst Evol Microbiol.

[19] Choo SW, Rishik S, Wee WY (2020). Comparative genome analyses of Mycobacteroides immunogenum reveals two potential novel subspecies. Microb Genom.

[20] Henssge U, Do T, Radford DR, Gilbert SC, Clark D, Beighton D (2009). Emended description of Actinomyces naeslundii and descriptions of Actinomyces oris sp. nov. and Actinomyces johnsonii sp. nov., previously identified as Actinomyces naeslundii genospecies 1, 2 and WVA 963. Int J Syst Evol Microbiol.

[21] Laing C, Buchanan C, Taboada EN, Zhang Y, Kropinski A, Villegas A (2010). Pan-genome sequence analysis using Panseq: an online tool for the rapid analysis of core and accessory genomic regions. BMC Bioinformatics..

[22] Alanjary M, Steinke K, Ziemert N (2019). AutoMLST: an automated web server for generating multi-locus species trees highlighting natural product potential. Nucleic Acids Res.

[23] Kumar S, Stecher G, Li M, Knyaz C, Tamura K (2018). MEGA X: Molecular Evolutionary Genetics Analysis across Computing Platforms. Mol Biol Evol..

[24] Stecher G, Tamura K, Kumar S (2020). Molecular evolutionary genetics analysis (MEGA) for macOS. Mol Biol Evol.

[25] Meier-Kolthoff JP, Carbasse JS, Peinado-Olarte RL, Goker M (2022). TYGS and LPSN: a database tandem for fast and reliable genome-based classification and nomenclature of prokaryotes. Nucleic Acids Res.

[26] Goris J, Konstantinidis KT, Klappenbach JA, Coenye T, Vandamme P, Tiedje JM (2007). DNA-DNA hybridization values and their relationship to whole-genome sequence similarities. Int J Syst Evol Microbiol.

[27] Meier-Kolthoff JP, Hahnke RL, Petersen J, Scheuner C, Michael V, Fiebig A (2014). Complete genome sequence of DSM 30083T, the type strain (U5/41T) of Escherichia coli, and a proposal for delineating subspecies in microbial taxonomy. Stand Genomic Sci..

[28] Deng W, Wang Y, Liu Z, Cheng H, Xue Y (2014). HemI: a toolkit for illustrating heatmaps. PLoS One.

[29] Rodriguez-R LM, Konstantinidis KT (2016). The enveomics collection: a toolbox for specialized analyses of microbial genomes and metagenomes. PeerJ Preprints. PeerJ Preprints.

[30] Rodriguez-R LM, Konstantinidis KT (2014). Bypassing Cultivation To Identify Bacterial Species: Culture-independent genomic approaches identify credibly distinct clusters, avoid cultivation bias, and provide true insights into microbial species. Microbe Magazine..

[31] Cabal A, Jun SR, Jenjaroenpun P, Wanchai V, Nookaew I, Wongsurawat T (2018). Genome-based comparison of Clostridioides difficile: average amino acid identity analysis of core genomes. Microb Ecol.

[32] Page AJ, Cummins CA, Hunt M, Wong VK, Reuter S, Holden MT (2015). Roary: rapid large-scale prokaryote pan genome analysis. Bioinformatics..

[33] Seemann T (2014). Prokka: rapid prokaryotic genome annotation. Bioinformatics.

[34] Cuccuru G, Orsini M, Pinna A, Sbardellati A, Soranzo N, Travaglione A (2014). Orione, a web-based framework for NGS analysis in microbiology. Bioinformatics..

[35] Hulsen T, de Vlieg J, Alkema W (2008). BioVenn - a web application for the comparison and visualization of biological lists using area-proportional Venn diagrams. BMC Genomics..

[36] Chaudhari NM, Gupta VK, Dutta C (2016). BPGA- an ultra-fast pan-genome analysis pipeline. Sci Rep..

[37] Xu L, Dong Z, Fang L, Luo Y, Wei Z, Guo H (2019). OrthoVenn2: a web server for whole-genome comparison and annotation of orthologous clusters across multiple species. Nucleic Acids Res..

[38] Sun J, Lu F, Luo Y, Bie L, Xu L, Wang Y (2023). OrthoVenn3: an integrated platform for exploring and visualizing orthologous data across genomes. Nucleic Acids Res.

[39] Nevers Y, Jones TEM, Jyothi D, Yates B, Ferret M, Portell-Silva L (2022). The Quest for Orthologs orthology benchmark service in 2022. Nucleic Acids Res.

[40] Bertelli C, Laird MR, Williams KP, Lau BY, Hoad G G (2017). IslandViewer 4: expanded prediction of genomic islands for larger-scale datasets. Nucleic Acids Res.

[41] Darling AC, Mau B, Blattner FR, Perna NT (2004). Mauve: multiple alignment of conserved genomic sequence with rearrangements. Genome Res.

[42] Cosentino S, Voldby Larsen M, Moller Aarestrup F, Lund O (2013). PathogenFinder–distinguishing friend from foe using bacterial whole genome sequence data. PLoS ONE..

[43] Liu B, Zheng D, Jin Q, Chen L, Yang J (2019). VFDB 2019: a comparative pathogenomic platform with an interactive web interface. Nucleic Acids Res.

[44] Liu B, Zheng D, Zhou S, Chen L, Yang J (2022). VFDB 2022: a general classification scheme for bacterial virulence factors. Nucleic Acids Res.

[45] Liu  L, Feng  Y, Wei  L, Zong  Z (2021). Genome-based taxonomy of brevundimonas with reporting brevundimonas huaxiensis sp. nov.. Microbiol Spectr.

[46] Couvin D, Bernheim A, Toffano-Nioche C, Touchon M, Michalik J, Neron B (2018). CRISPRCasFinder, an update of CRISRFinder, includes a portable version, enhanced performance and integrates search for Cas proteins. Nucleic Acids Res.

[47] Hitch TCA, Riedel T, Oren A, Overmann J, Lawley TD, Clavel T (2021). Automated analysis of genomic sequences facilitates high-throughput and comprehensive description of bacteria. ISME Commun.

[48] Oren A, Arahal DR, Göker M, Moore ERB, Rossello-Mora R, Sutcliffe IC. International Code of Nomenclature of Prokaryotes. Prokaryotic Code (2022 Revision). Int J Syst Evol Microbiol. 2023;73(5a)10–30. 10.1099/ijsem.0.005585 37219928

[49] Fujimoto A, Wong JH, Yoshii Y, Akiyama S, Tanaka A, Yagi H (2021). Whole-genome sequencing with long reads reveals complex structure and origin of structural variation in human genetic variations and somatic mutations in cancer. Genome Medicine.

[50] Auch AF, von Jan M, Klenk HP, Goker M (2010). Digital DNA-DNA hybridization for microbial species delineation by means of genome-to-genome sequence comparison. Stand Genomic Sci..

[51] Shamsuzzaman M, Dahal RH, Kim S, Kim J (2023). Genome insight and probiotic potential of three novel species of the genus Corynebacterium. Front Microbiol..

[52] Khoder M, Osman M, Kassem II, Rafei R, Shahin A, Fournier PE (2022). Whole genome analyses accurately identify Neisseria spp. and limit taxonomic ambiguity. Int J Mol Sci.

[53] Viver T, Conrad RE, Rodriguez-R LM, Ramírez AS, Venter SN, Rocha-Cárdenas J (2024). Towards estimating the number of strains that make up a natural bacterial population. Nat Commun..

[54] Kim E, Yang SM, Kim IS, Lee SY, Kim HY (2022). Identification of Leuconostoc species based on novel marker genes identified using real-time PCR via computational pangenome analysis. Front Microbiol..

[55] Reimer LC, Sardà Carbasse J, Koblitz J, Ebeling C, Podstawka A, Overmann J (2021). BacDive in 2022: the knowledge base for standardized bacterial and archaeal data. Nucleic Acids Res..

[56] Tambong JT, Xu R, Gerdis S, Daniels GC, Chabot D, Hubbard K (2021). Molecular analysis of bacterial isolates from necrotic wheat leaf lesions caused by xanthomonas translucens, and description of three putative novel species, sphingomonas albertensis sp. nov., Pseudomonas triticumensis sp. nov. and Pseudomonas foliumensis sp. nov.. Front Microbiol.

[57] Wee WY, Chew XY, Taheri S, Tan XL, Teo CH (2022). Whole genome sequencing and phylogenomic analyses of a novel glufosinate-tolerant Pseudomonas species. Biotech.

[58] Topaz N, Boxrud D, Retchless AC, Nichols M, Chang H-Y, Hu F (2018). BMScan: using whole genome similarity to rapidly and accurately identify bacterial meningitis causing species. BMC Infect Dis.

[59] Volpiano CG, Sant’Anna FH, Ambrosini A, de São José JFB, Beneduzi A, Whitman WB, et al. Genomic Metrics Applied to Rhizobiales (Hyphomicrobiales): Species Reclassification, Identification of Unauthentic Genomes and False Type Strains. Front Microbiol. 2021;12:1–2. 10.3389/fmicb.2021.614957 PMC8026895 33841347

[60] Oliphant SA, Watson-Haigh NS, Sumby KM, Gardner J, Groom S, Jiranek V (2022). Apilactobacillus apisilvae sp. nov., Nicolia spurrieriana gen. nov. sp. nov., Bombilactobacillus folatiphilus sp. nov. and Bombilactobacillus thymidiniphilus sp. nov., four new lactic acid bacterial isolates from stingless bees Tetragonula carbonaria and Austroplebeia australis. Int J Syst Evol Microbiol.

[61] Richter M, Rossello-Mora R (2009). Shifting the genomic gold standard for the prokaryotic species definition. Proc Natl Acad Sci U S A.

[62] Chun J, Oren A, Ventosa A, Christensen H, Arahal DR, da Costa MS (2018). Proposed minimal standards for the use of genome data for the taxonomy of prokaryotes. Int J Syst Evol Microbiol.

[63] Riesco R, Trujillo ME (2024). Update on the proposed minimal standards for the use of genome data for the taxonomy of prokaryotes. Int J Syst Evol Microbiol.

[64] Jiang S, Fan Q, Zhang Z, Deng Y, Wang L, Dai Q (2023). Biodegradation of Oil by a Newly Isolated Strain Acinetobacter junii WCO-9 and Its Comparative Pan-Genome Analysis. Microorganisms.

[65] Zhong C, Qu B, Hu G, Ning K (2022). Pan-genome analysis of campylobacter: insights on the genomic diversity and virulence profile. Microbiol Spectr.

[66] Kim Y, Koh I, Young Lim M, Chung WH, Rho M (2017). Pan-genome analysis of Bacillus for microbiome profiling. Sci Rep.

[67] Svetlicic E, Jaén-Luchoro D, Klobucar RS, Jers C, Kazazic S, Franjevic D (2023). Genomic characterization and assessment of pathogenic potential of Legionella spp. isolates from environmental monitoring. Front Microbiol.

[68] Sitto F, Battistuzzi FU (2020). Estimating pangenomes with roary. Mol Biol Evol.

[69] Le DQ, Nguyen TA, Nguyen TT, Nguyen SH, Do VH, Nguyen CH, et al. PanTA: An ultra-fast method for constructing large and growing microbial pangenomes. bioRxiv. 2023:2023.07.03.547471. 10.1101/2023.07.03.547471.

[70] Enright AJ, Van Dongen S, Ouzounis CA (2002). An efficient algorithm for large-scale detection of protein families. Nucleic Acids Res.

[71] Eskova AI, Andryukov BG, Yakovlev AA, Kim AV, Ponomareva AL, Obuhova VS (2022). Horizontal transfer of virulence factors by pathogenic enterobacteria to marine saprotrophic bacteria during co-cultivation in biofilm. Biotech..

[72] Li W, Wang A (2021). Genomic islands mediate environmental adaptation and the spread of antibiotic resistance in multiresistant Enterococci - evidence from genomic sequences. BMC Microbiol.

[73] Saini A, Mani I, Rawal MK, Verma C, Singh V, Mishra SK, Mani I, Singh V, Alzahrani KJ, Chu D-T (2023). An Introduction to Microbial Genomic Islands for Evolutionary Adaptation and Pathogenicity. Microbial Genomic Islands in Adaptation and Pathogenicity.

[74] Roggenkamp A, Bittner T, Leitritz L, Sing A, Heesemann J (1997). Contribution of the Mn-cofactored superoxide dismutase (SodA) to the virulence of Yersinia enterocolitica serotype O8. Infect Immun.

[75] Poyart C, Pellegrini E, Gaillot O, Boumaila C, Baptista M, Trieu-Cuot P (2001). Contribution of Mn-Cofactored Superoxide Dismutase (SodA) to the Virulence of *Streptococcus agalactiae*. Infect Immun.

[76] Qu H, Xin Y, Dong X, Ma Y (2007). An rmlA gene encoding d-glucose-1-phosphate thymidylyltransferase is essential for mycobacterial growth. FEMS Microbiol Lett.

[77] Pföstl A, Zayni S, Hofinger A, Kosma P, Schäffer C, Messner P (2008). Biosynthesis of dTDP-3-acetamido-3,6-dideoxy-α-D-glucose. Biochemical Journal.

[78] Barrangou R, Marraffini LA (2014). CRISPR-Cas systems: Prokaryotes upgrade to adaptive immunity. Mol Cell.

[79] Makarova KS, Wolf YI, Iranzo J, Shmakov SA, Alkhnbashi OS, Brouns SJJ (2020). Evolutionary classification of CRISPR-Cas systems: a burst of class 2 and derived variants. Nat Rev Microbiol.

[80] Yoshimi K, Takeshita K, Kodera N, Shibumura S, Yamauchi Y, Omatsu M (2022). Dynamic mechanisms of CRISPR interference by Escherichia coli CRISPR-Cas3. Nat Commun.

[81] Boer Nt, editor. Spacer integration by the Cas 1-Cas 2 protein complex in different CRISPR-Cas systems. 2018.

[82] Arslan Z, Hermanns V, Wurm R, Wagner R, Pul Ü (2014). Detection and characterization of spacer integration intermediates in type I-E CRISPR–Cas system. Nucleic Acids Res.

