# Supplementary material for: Genome characterisation and comparative analysis of Schaalia dentiphila sp. nov. and its subspecies, S. dentiphila subsp. denticola subsp. nov., from the human oral cavity
Source: BMC Microbiol. 2024 May 28;24:185. doi: 10.1186/s12866-024-03346-w (PMC11131293; doi:10.1186/s12866-024-03346-w)
Supplement: Supplementary file 1 — Supplementary Material 1. [file 12866_2024_3346_MOESM1_ESM.docx]

**Supplementary Information**

Xuechen Tian^1,2,3,4^, Wee Fei Aaron Teo^1^, Yixin Yang^2,3,4,5^, Linyinxue Dong^3,4^, Aloysius Wong^2,3,4,5^, Li Chen^1^, Halah Ahmed^6^, Siew Woh Choo^2,3,^^4,5*^, Nicholas S. Jakubovics^6*^ and Geok Yuan Annie Tan^1*^

^1^Institute of Biological Sciences, Faculty of Science, Universiti Malaya, 50603 Kuala Lumpur, Malaysia.

^2^College of Science, Mathematics and Technology, Wenzhou-Kean University, 88 Daxue Road, Ouhai, Wenzhou, Zhejiang Province, 325060 China.

^3^Wenzhou Municipal Key Laboratory for Applied Biomedical and Biopharmaceutical Informatics, Wenzhou-Kean University, Ouhai, Wenzhou, Zhejiang Province, 325060 China.

^4^Zhejiang Bioinformatics International Science and Technology Cooperation Center, Wenzhou-Kean University, Ouhai, Wenzhou, Zhejiang Province, 325060 China.

^5^Dorothy and George Hennings College of Science, Mathematics and Technology, Kean University, 1000 Morris Ave, Union, NJ 07083, USA.

^6^School of Dental Sciences, Faculty of Medical Sciences, Newcastle University, Framlington Place, Newcastle upon Tyne, NE2 4BW, UK.

***Corresponding authors:**

Geok Yuan Annie Tan; Email: [gyatan@um.edu.my](mailto:gyatan@um.edu.my); Institute of Biological Sciences, Faculty of Science, Universiti Malaya, 50603 Kuala Lumpur, Malaysia.

Nicholas S. Jakubovics; Email: [nick.jakubovics@newcastle.ac.uk](mailto:nick.jakubovics@newcastle.ac.uk); School of Dental Sciences, Faculty of Medical Sciences, Newcastle University, Framlington Place, Newcastle upon Tyne, NE2 4BW, UK.

Siew Woh Choo; Email: [cwoh@wku.edu.cn](mailto:cwoh@wku.edu.cn); College of Science, Mathematics and Technology, Wenzhou-Kean University, 88 Daxue Road, Ouhai, Wenzhou, Zhejiang Province, 325060 China.


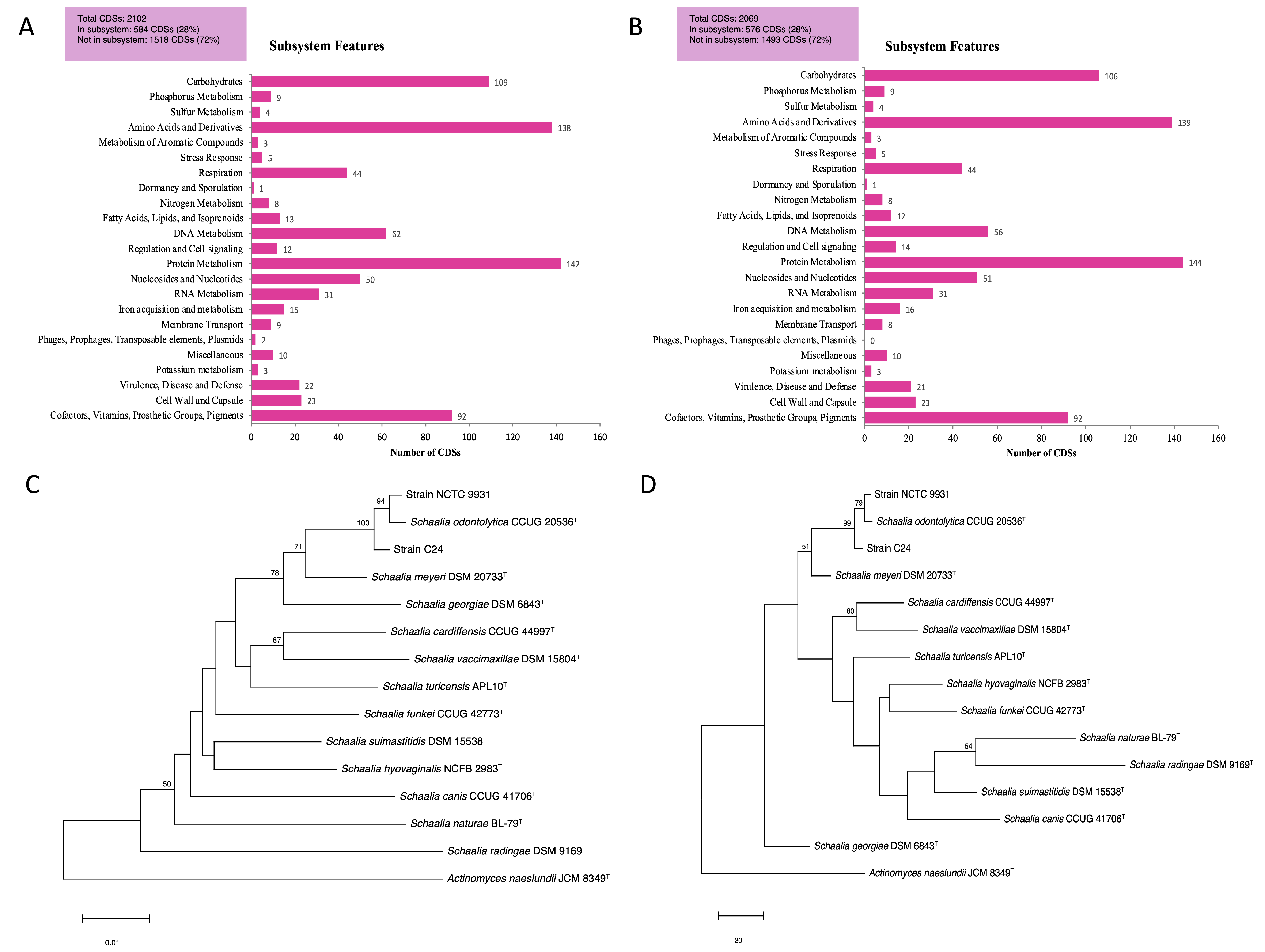


**Figure S1. Genome annotation and 16S rRNA phylogenetic relationship.** (A) Subsystem features distribution associated with the protein-coding gene of strain NCTC 9931 by RAST annotation. (B) Subsystem features distribution associated with the protein-coding gene of strain C24 by RAST annotation. (C) A neighbor-joining phylogenetic tree based on 16S rRNA gene sequences of strains NCTC 9931 and C24 compared to the type strains of *Schaalia* species, the tree was inferred using the neighbor-joining method with *Kimura* 2-parameter model. (D) A maximum parsimony phylogenetic tree based on 16S rRNA gene sequences of strains NCTC 9931 and C24 compared to type strains of *Schaalia* species, the tree was inferred using the maximum parsimony method with subtree-pruning-regrafting (SPR) model. *Actinomyces naeslundii* JCM 8349^T^ was employed as an outgroup in two trees. Bootstrap value (percentage) was computed based on 1,000 bootstrap replicates, and values with more than 50% are shown. Note that *Schaalia odontolytica* CCUG 20536^T^ and NCTC 9935^T^ are synonyms of the same strain.


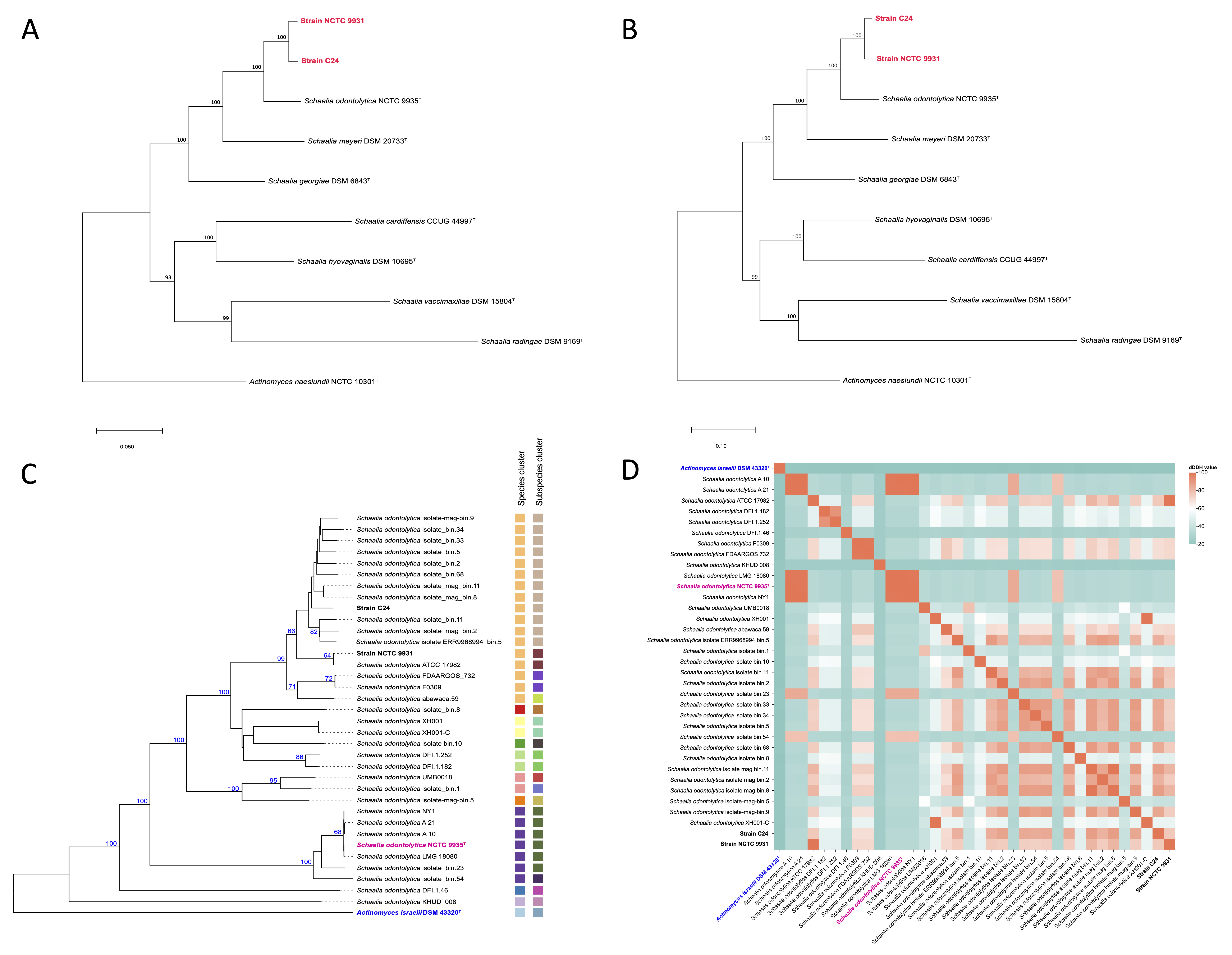
**Figure S2**. **Multiple genes phylogenetic analysis and type stain genome server (TYGS) analysis.** (A) Phylogenomic tree constructed using five housekeeping gene sequences (*atpA, rpoB, pgi, metG, and gyrA)* extracted from the genomes of strains NCTC 9931 and C24, along with seven available *Schaalia* type strains. *Actinomyces naeslundii* NCTC 10301^T^ was employed as an outgroup. The tree was constructed using the maximum-likelihood method with 1,000 bootstrap replicates, and bootstrap values above 50% are shown. (B) Phylogenomic tree based on the concatenated nucleotide sequences of core genome SNPs sequences extracted from the genomes of strains NCTC 9931 and C24, as well as seven available *Schaalia* type strains. *Actinomyces naeslundii* NCTC 10301^T^ was employed as an outgroup. The tree was constructed using the maximum-likelihood method with 1,000 bootstrap replicates, and bootstrap values above 50% are shown. (C) Genome sequence-based tree from TYGS analysis results for two strains and 33 *Schaalia odontolytica* assemblies. Species cluster denotes groupings formed using a 70% dDDH threshold, and subspecies cluster indicates groupings established with a more stringent 79% dDDH threshold. *Actinomyces israelii* DSM 43320^T^ was employed as an outgroup. (D) Heatmap showing dDDH values between two analyzed strains and 33 *Schaalia odontolytica* assemblies. These dDDH values were calculated based on the confidence interval of formula *d_4_* in GBDP. *Actinomyces israelii* DSM 43320^T^ was employed as an outgroup. Type strain assemblies are highlighted in red and blue, and all genome assembly information of *Schaalia odontolytica* can be accessed in **Table S2**.


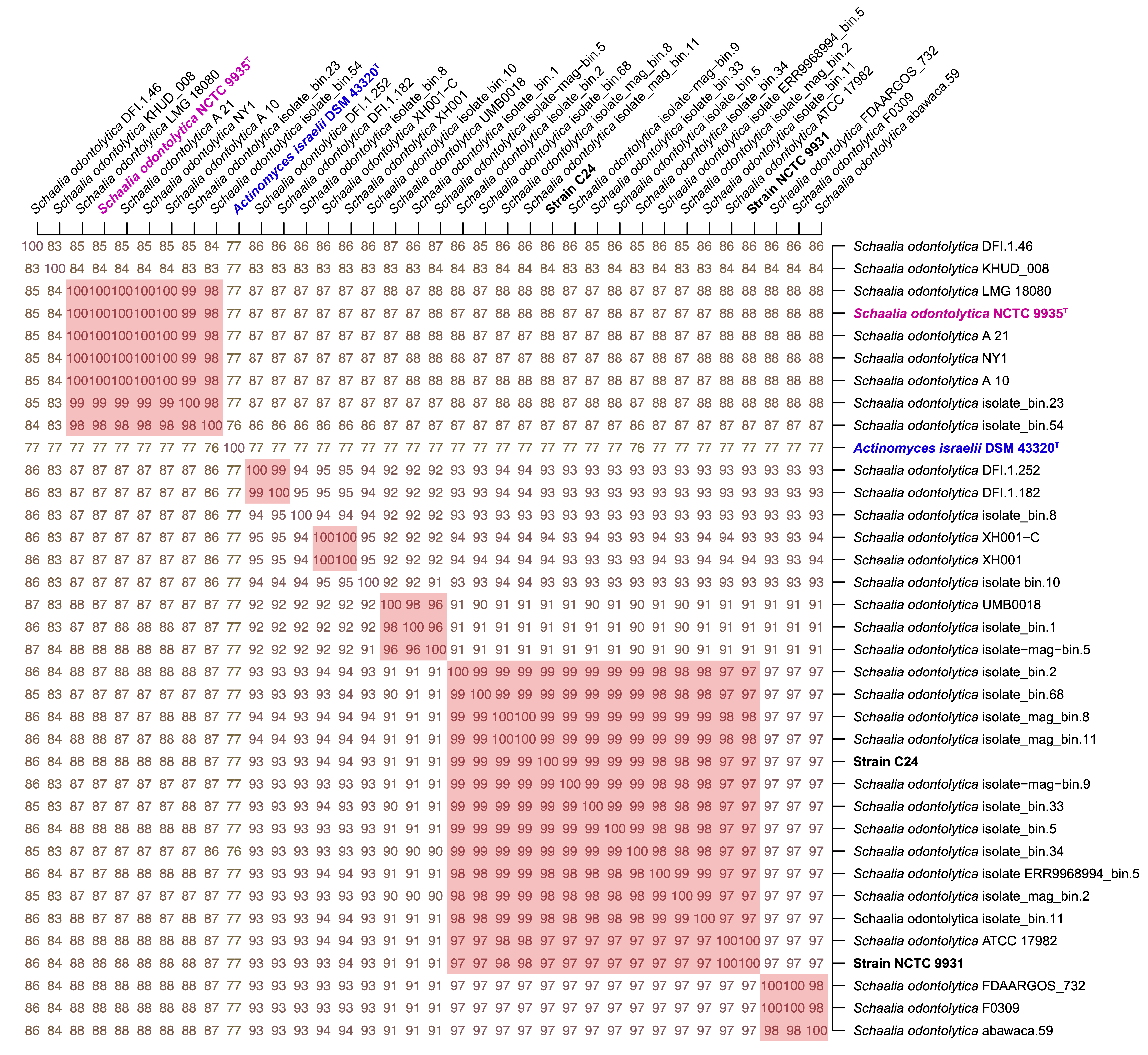


**Figure S3. ANI analysis of two analyzed strains and 33 available *Schaalia odontolytica* assemblies, *Actinomyces israelii* DSM 43320^T^ was employed as an outgroup.** This matrix displays the ANI values between strains NCTC 9931 and C24, and various *Schaalia odontolytica* assemblies. ANI values greater than 96% are marked by a red background, suggesting a high genomic similarity indicative of species-level relationships. Type strain assemblies are distinguished by being highlighted in red and blue.


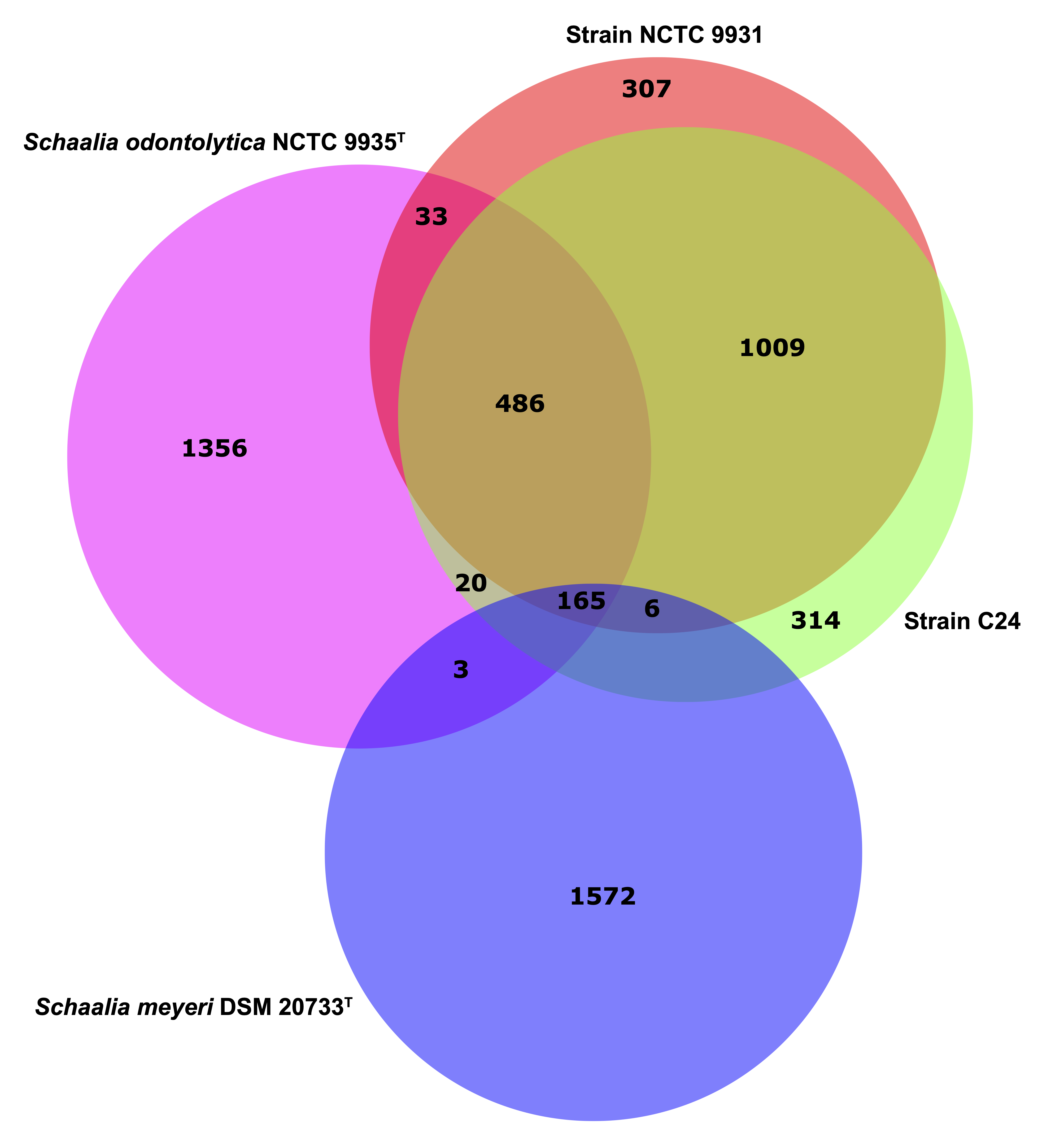


**Figure S4.** **Venn diagram showing the numbers of core, accessory, and unique genes present in four selected strains using the Roary pipeline.** The four selected strains are strain NCTC 9931, strain C24, *Schaalia odontolytica* NCTC 9935^T^, and *Schaalia meyeri* DSM 20733^T^.

**Figure S5. Analysis of the pangenome annotations.** (A) Distribution of Clusters of Orthologous Groups of Proteins (COGs) across the pangenome of the four strains. (B) Distribution of KEGG pathways in the pangenome of the four strains. The strains analyzed include strain NCTC 9931, strain C24, *Schaalia odontolytica* NCTC 9935^T^, and *Schaalia meyeri* DSM 20733^T^.

**Figure S6. GO enrichment analysis of expanded and contracted gene families of strains NCTC 9931 and C24.** (A) GO distribution of 21 expanded gene families in strain C24, the red bar indicates the molecular function, and the blue bar represents the biological process. (B) GO distribution of 11 expanded gene families in strain NCTC 9931, and the blue bar represents the biological process. (C) GO distribution of 19 contracted gene families in strain C24, the violet bar indicates the cellular component, and the blue bar represents the biological process. (D) GO distribution of 23 contracted gene families in strain NCTC 9931, and the blue bar represents the biological process.


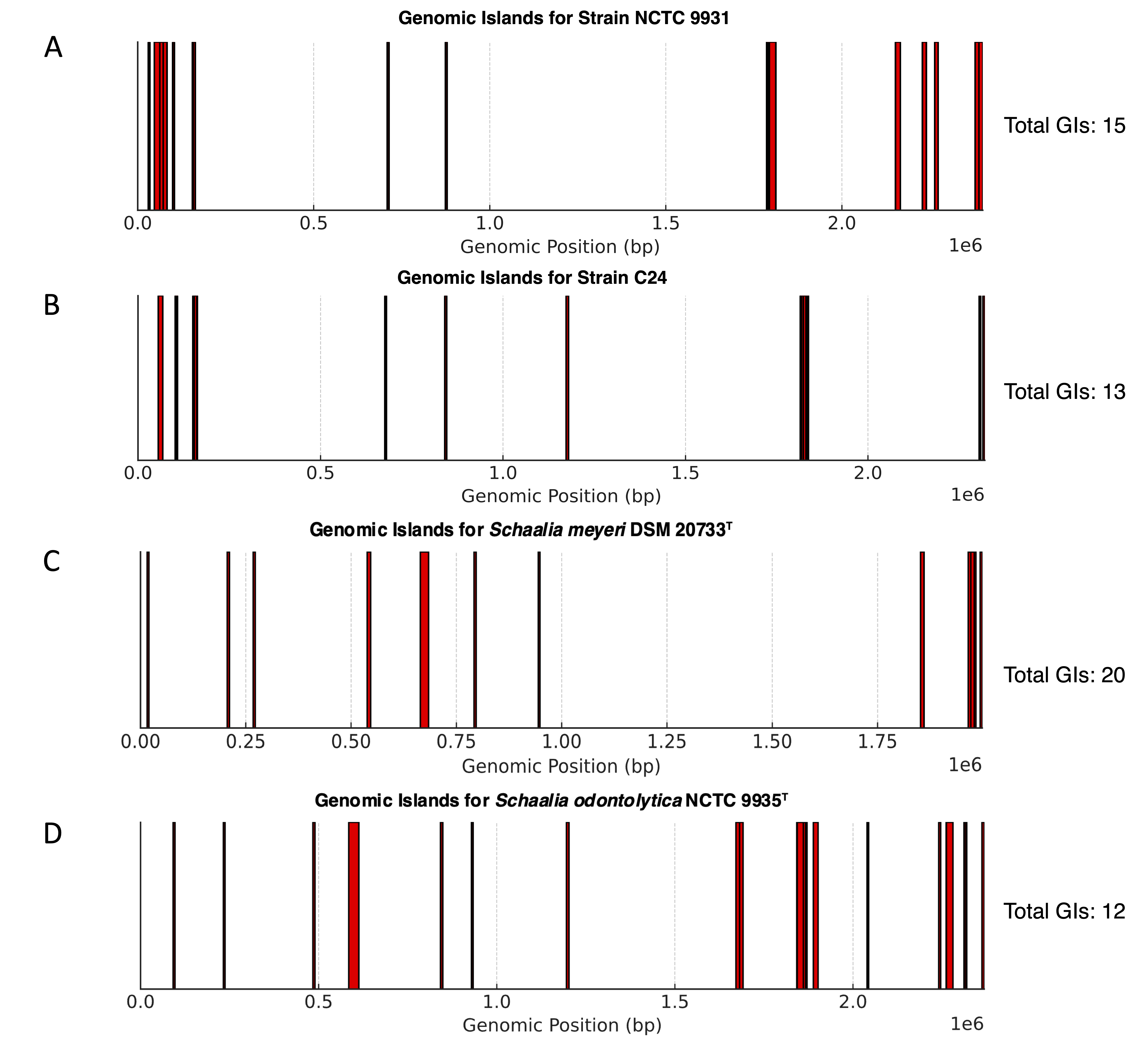


**Figure S7. Genomic islands (GIs) distribution for four strains**. (A) GIs distribution of strain NCTC 9931. (B) GIs distribution of strain C24. (C) GIs distribution of strains of *Schaalia odontolytica* NCTC 9935^T^. (D) GIs distribution of strains *Schaalia meyeri* DSM 20733^T^. The colored rectangles represent GIs, and the larger the rectangle, the larger the GIs.


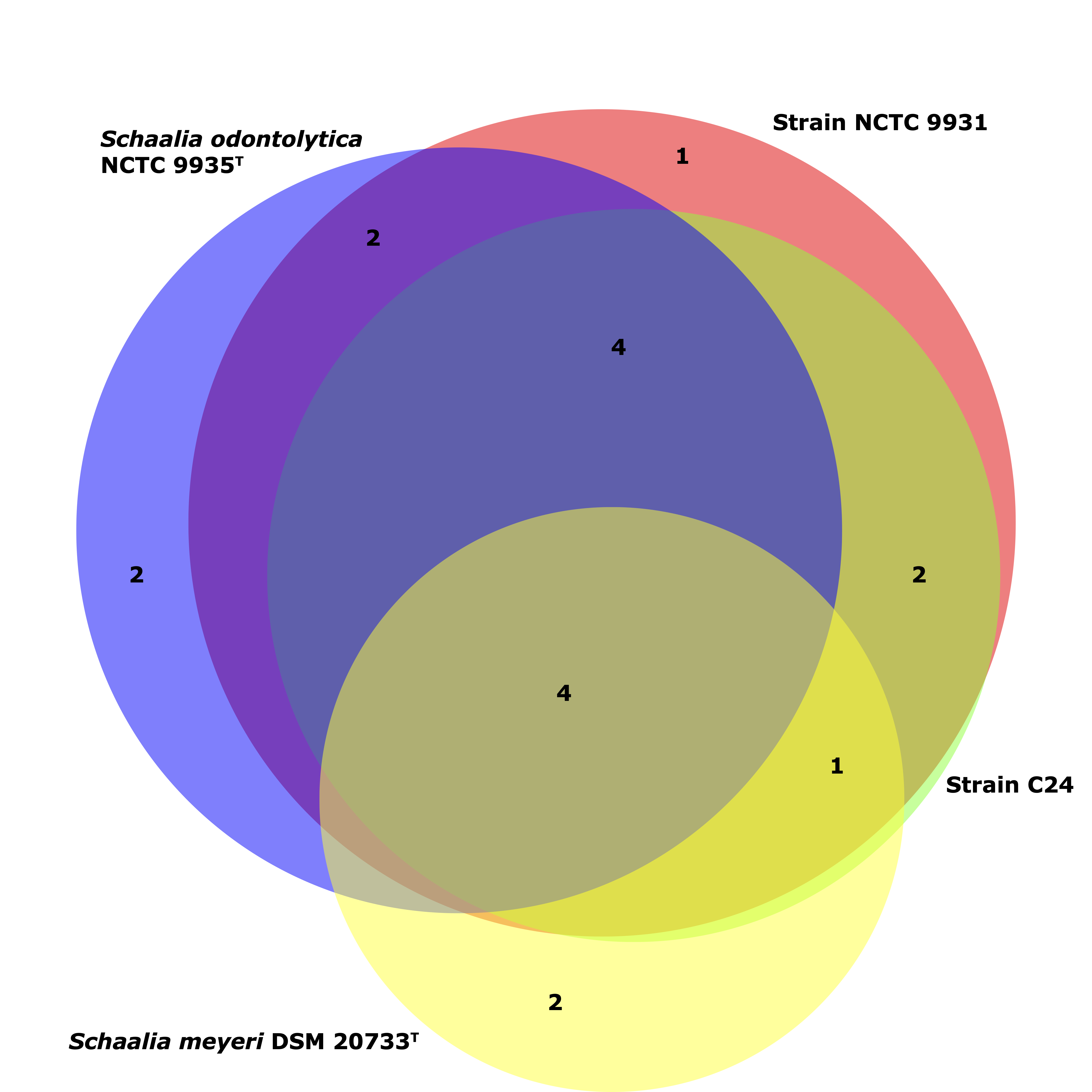


**Figure S8. Venn diagram showing the numbers of virulence factors for four selected strains.** The four selected strains are strain NCTC 9931, strain C24, *Schaalia odontolytica* NCTC 9935^T^, and *Schaalia meyeri* DSM 20733^T^. Detailed information is presented in Table 3.

**Table S1. Genome information and quality assessment used in this study, these genomes were retracted from the RefSeq database in NCBI.**

| **No** | **Strain Name** | **Genome Accession Number** | **Genome Size (bp)** | **Completeness (%)** | **Contamination (%)** | **GC Content (%)** |
| --- | --- | --- | --- | --- | --- | --- |
| 1 | *Schaalia odontolytica* NCTC 9935^T^ | GCF_900445025.1 | 2,454,426 | 99.76 | 0.95 | 64.86 |
| 2 | *Schaalia meyeri* DSM 20733^T^ | GCF_900105015.1 | 2,026,407 | 97.63 | 2.18 | 65.58 |
| 3 | *Schaalia georgiae* DSM 6843^T^ | GCF_000429245.1 | 2,498,023 | 100 | 0.47 | 69.87 |
| 4 | *Schaalia cardiffensis* CCUG 44997^T^ | GCF_016405145.1 | 2,240,847 | 100 | 3.91 | 61.57 |
| 5 | *Schaalia hyovaginalis* DSM 10695^T^ | GCF_014208035.1 | 2,647,349 | 100 | 1.90 | 67.24 |
| 6 | *Schaalia vaccimaxillae* DSM 15804^T^ | GCF_000420425.1 | 2,338,426 | 100 | 0.47 | 57.56 |
| 7 | *Schaalia radingae* DSM 9169^T^ | GCF_900106055.1 | 2,430,293 | 99.29 | 2.84 | 58.93 |
| 8 | Strain NCTC 9931 | JASPFE000000000 | 2,374,847 | 100 | 0.47 | 65.49 |
| 9 | Strain C24 | JASPEY000000000 | 2,345,519 | 100 | 0.47 | 65.47 |
| 10 | *Actinomyces naeslundii* NCTC 10301^T^ | GCF_001956585.1 | 3,119,690 | 100 | 0.47 | 67.94 |

**Table S2. Genome assemblies of *Schaalia odontolytica* and outgroup *Actinomyces israelii* DSM 43320^T^ available in NCBI. These genomes were used for genome comparative analysis.**

| **No** | **Strain Name** | **Genome Accession Number** | **Genome Size (Mp)** | **Completeness (%)** | **Contamination (%)** | **GC Content (%)** |
| --- | --- | --- | --- | --- | --- | --- |
| 1 | *Schaalia odontolytica* ATCC 17982 | GCA_000154225.1 | 2.4 | 100 | 0 | 65.5 |
| 2 | *Schaalia odontolytica* F0309 | GCA_000163415.1 | 2.4 | 100 | 0.47 | 65 |
| 3 | *Schaalia odontolytica* XH001 | GCA_001462375.1 | 2.3 | 100 | 0.47 | 66 |
| 4 | *Schaalia odontolytica* UMB0018 | GCA_002847525.1 | 2.3 | 100 | 0.47 | 65.5 |
| 5 | *Schaalia odontolytica* XH001-1 | GCA_005696695.1 | 2.4 | 99.41 | 0.47 | 66 |
| 6 | *Schaalia odontolytica* FDAARGOS_732 | GCA_009730335.1 | 2.4 | 99.91 | 0.47 | 65.5 |
| 7 | *Schaalia odontolytica* DFI.1.46 | GCA_020561905.1 | 2.5 | 100 | 0.47 | 67 |
| 8 | *Schaalia odontolytica* DFI.1.252 | GCA_024463675.1 | 2.4 | 91.39 | 0.47 | 65.5 |
| 9 | *Schaalia odontolytica* DFI.1.182 | GCA_024463835.1 | 2.3 | 91.23 | 0.47 | 66 |
| 10 | *Schaalia odontolytica* KHUD_008 | GCA_024584435.1 | 2.4 | 98.82 | 0.95 | 66.5 |
| 11 | *Schaalia odontolytica* NCTC 9935^T^ | GCA_900445025.1 | 2.5 | 99.05 | 0.47 | 65 |
| 12 | *Schaalia odontolytica* LMG 18080 | GCA_031191545.1 | 2.5 | 99.91 | 0.47 | 65 |
| 13 | *Schaalia odontolytica* NY1 | GCA_031296445.1 | 2.4 | 99.91 | 0.47 | 65 |
| 14 | *Schaalia odontolytica* A 10 | GCA_031296555.1 | 2.4 | 99.91 | 0.47 | 65 |
| 15 | *Schaalia odontolytica* A 21 | GCA_031296525.1 | 2.4 | 99.91 | 0.47 | 65 |
| 16 | *Schaalia odontolytica* isolate_mag_bin.5 | GCA_905369815.1 | 1.9 | NA | NA | 66 |
| 17 | *Schaalia odontolytica* isolate_mag_bin.9 | GCA_905373765.1 | 2.3 | 97.11 | 1.5 | 65.5 |
| 18 | *Schaalia odontolytica* isolate_bin.2 | GCA_916049885.1 | 2.4 | 99.07 | 0.47 | 65.5 |
| 19 | *Schaalia odontolytica* isolate_bin.5 | GCA_927911795.1 | 2 | NA | NA | 65 |
| 20 | *Schaalia odontolytica* isolate_bin.54 | GCA_938041085.1 | 2.2 | NA | NA | 65.5 |
| 21 | *Schaalia odontolytica* isolate_bin.68 | GCA_938046745.1 | 2.4 | NA | NA | 65.5 |
| 22 | *Schaalia odontolytica* isolate_bin.10 | GCA_963521265.1 | 2.3 | 99.53 | 0.47 | 66 |
| 23 | *Schaalia odontolytica* isolate ERR9968994_bin.5 | GCA_963525615.1 | 2.3 | 99.91 | 0.47 | 65.5 |
| 24 | *Schaalia odontolytica* isolate_bin.23 | GCA_963519065.1 | 2.4 | 99.05 | 0.95 | 65 |
| 25 | *Schaalia odontolytica* isolate_bin.8 | GCA_963546915.1 | 2.2 | 94.5 | 0.47 | 66 |
| 26 | *Schaalia odontolytica* isolate_bin.1 | GCA_963524845.1 | 2.1 | 95.36 | 0.57 | 66 |
| 27 | *Schaalia odontolytica* isolate_bin.34 | GCA_963550835.1 | 2.2 | 91.55 | 1.48 | 65.5 |
| 28 | *Schaalia odontolytica* isolate_bin.11 | GCA_963554195.1 | 2.3 | 94.72 | 1.46 | 65.5 |
| 29 | *Schaalia odontolytica* isolate_bin.33 | GCA_963549115.1 | 2.2 | 91.66 | 0.98 | 65.5 |
| 30 | *Schaalia odontolytica* isolate_mag_bin.8 | GCA_963507435.1 | 2 | 93.11 | 0.71 | 66 |
| 31 | *Schaalia odontolytica* isolate_mag_bin.11 | GCA_963531195.1 | 2 | 93.11 | 0.71 | 66 |
| 32 | *Schaalia odontolytica* abawaca.59 | GCA_032560095.1 | 2.3 | 97.1 | 1.37 | 65.5 |
| 33 | *Schaalia odontolytica* isolate_mag_bin.2 | GCA_963456885.1 | 2 | NA | NA | 65.5 |
| 34 | *Actinomyces israelii* DSM 43320^T^ | GCA_000711965.1 | 4 | 99.89 | 0.23 | 71.5 |

**Table S3. Number of proteins, clusters, and singletons generated by OrthoVenn3 analysis.**

| **Strain** | **Proteins** | **Clusters** | **Singletons** |
| --- | --- | --- | --- |
| Strain NCTC 9931 | 2,102 | 1,896 | 86 |
| Strain C24 | 2,069 | 1,881 | 67 |
| *Schaalia odontolytica* NCTC 9935^T^ | 2,132 | 1,838 | 155 |
| *Schaalia meyeri DSM* 20733^T^ | 1,794 | 1,564 | 106 |

**Table S4. The genomic islands (GIs) distribution statistic of four strains**

| **Strain** | **GIs** | **Island start** | **Island end** | **Length (bp)** | **Gene number** |
| --- | --- | --- | --- | --- | --- |
| Strain NCTC 9931 | GI1 | 30,003 | 34,485 | 4,482 | 5 |
|  | GI2 | 47,333 | 82,825 | 35,492 | 30 |
|  | GI3 | 62,717 | 72,043 | 9,326 | 11 |
|  | GI4 | 99,222 | 103,980 | 4,758 | 5 |
|  | GI5 | 155,188 | 162,105 | 6,917 | 8 |
|  | GI6 | 157,758 | 163,538 | 5,780 | 7 |
|  | GI7 | 708,887 | 713,788 | 4,901 | 7 |
|  | GI8 | 874,168 | 878,978 | 4,810 | 7 |
|  | GI9 | 1,786,108 | 1,812,105 | 25,997 | 23 |
|  | GI10 | 1,789,800 | 1,793,968 | 4,168 | 2 |
|  | GI11 | 2,152,312 | 2,166,171 | 13,859 | 11 |
|  | GI12 | 2,228,898 | 2,239,702 | 10,804 | 21 |
|  | GI13 | 2,263,710 | 2,273,269 | 9,559 | 12 |
|  | GI14 | 2,378,449 | 2,394,744 | 16,295 | 11 |
|  | GI15 | 2,389,268 | 2,400,056 | 10,788 | 8 |
| Strain C24 | GI1 | 55,722 | 67,855 | 12,133 | 11 |
|  | GI2 | 101,978 | 108,253 | 6,275 | 8 |
|  | GI3 | 103,087 | 107,409 | 4,322 | 6 |
|  | GI4 | 150,350 | 162,352 | 12,002 | 12 |
|  | GI5 | 154,070 | 160,924 | 6,854 | 9 |
|  | GI6 | 676,320 | 680,525 | 4,205 | 6 |
|  | GI7 | 840,761 | 845,618 | 4,857 | 7 |
|  | GI8 | 1,172,771 | 1,179,285 | 6,514 | 3 |
|  | GI9 | 1,814,910 | 1,836,894 | 21,984 | 20 |
|  | GI10 | 1,817,879 | 1,822,916 | 5,037 | 6 |
|  | GI11 | 1,830,518 | 1,834,668 | 4,150 | 4 |
|  | GI12 | 2,304,782 | 2,308,856 | 4,074 | 5 |
|  | GI13 | 2,315,139 | 2,320,129 | 4,990 | 5 |
| *Schaalia odontolytica* NCTC 9935^T^ | GI1 | 91,722 | 96,787 | 5,065 | 4 |
|  | GI2 | 232,718 | 237,403 | 4,685 | 4 |
|  | GI3 | 483,732 | 489,472 | 5,740 | 6 |
|  | GI4 | 584,515 | 612,884 | 28,369 | 24 |
|  | GI5 | 842,062 | 848,468 | 6,406 | 8 |
|  | GI6 | 929,230 | 933,996 | 4,766 | 5 |
|  | GI7 | 1,195,831 | 1,203,004 | 7,173 | 3 |
|  | GI8 | 1,671,634 | 1,686,529 | 14,895 | 16 |
|  | GI9 | 1,681,671 | 1,691,603 | 9,932 | 17 |
|  | GI10 | 1,842,153 | 1,852,039 | 9,886 | 16 |
|  | GI11 | 1,843,295 | 1,870,910 | 27,615 | 33 |
|  | GI12 | 1,860,905 | 1,867,367 | 6,462 | 9 |
|  | GI13 | 1,888,823 | 1,902,150 | 13,327 | 12 |
|  | GI14 | 2,039,680 | 2,043,888 | 4,208 | 4 |
|  | GI15 | 2,240,523 | 2,246,184 | 5,661 | 6 |
|  | GI16 | 2,261,398 | 2,278,272 | 16,874 | 21 |
|  | GI17 | 2,262,169 | 2,280,287 | 18,118 | 21 |
|  | GI18 | 2,311,490 | 2,318,936 | 7,446 | 8 |
|  | GI19 | 2,312,600 | 2,317,119 | 4,519 | 5 |
|  | GI20 | 2,362,232 | 2,368,475 | 6,243 | 6 |
| *Schaalia meyeri* DSM 20733^T^ | GI1 | 15,915 | 20,271 | 4,356 | 7 |
|  | GI2 | 206,112 | 211,582 | 5,470 | 4 |
|  | GI3 | 267,473 | 272,523 | 5,050 | 6 |
|  | GI4 | 538,457 | 546,605 | 8,148 | 11 |
|  | GI5 | 664,539 | 684,278 | 19,739 | 22 |
|  | GI6 | 664,539 | 671,620 | 7,081 | 12 |
|  | GI7 | 791,909 | 796,834 | 4,925 | 3 |
|  | GI8 | 944,677 | 948,736 | 4,059 | 4 |
|  | GI9 | 1,852,183 | 1,860,346 | 8,163 | 6 |
|  | GI10 | 1,965,556 | 1,983,971 | 18,415 | 21 |
|  | GI11 | 1,971,578 | 1,979,896 | 8,318 | 12 |
|  | GI12 | 1,993,806 | 1,998,729 | 4,923 | 5 |

**Table S5.** **Pathogenic analysis results using the PathogenFinder online tool.**

| **Strain** | **Strain NCTC 9931** | **Strain C24** |
| --- | --- | --- |
| Human pathogen | Yes | Yes |
| Matched Family | 1 | 1 |
| Organisms | *Bifidobacterium dentium* Bd1 | *Bifidobacterium dentium* Bd1 |
| Class | *Actinomycetes* *(Actinobacteridae* subclass) | *Actinomycetes* *(Actinobacteridae* subclass) |
| Protein function | Conserved hypothetical protein | Conserved hypothetical protein |
| Identity (%) | 87.50% | 85.50% |

**Table S6. CRISPR distribution of four strains**

| **Strain** | **Element** | **Start** | **End** | **CRISPR Length** | **Repeat Length** | **Spacer count** |
| --- | --- | --- | --- | --- | --- | --- |
| Strain NCTC 9931 | CRISPR | 224,104 | 224,214 | 110 | 27 | 1 |
|  | CRISPR | 302,872 | 303,078 | 206 | 27 | 3 |
|  | CRISPR | 174,358 | 174,446 | 88 | 28 | 1 |
|  | CRISPR | 140,482 | 140,822 | 340 | 23 | 6 |
|  | CRISPR | 32,882 | 32,956 | 74 | 23 | 1 |
|  | CRISPR | 64,457 | 64,545 | 88 | 32 | 1 |
|  | CRISPR | 57,622 | 57,739 | 117 | 38 | 1 |
|  | CRISPR | 29,511 | 36,984 | 7,473 | 28 | 122 |
|  | CRISPR | 4,081 | 4,287 | 206 | 24 | 3 |
| Strain C24 | CRISPR | 62,709 | 62,794 | 85 | 29 | 1 |
|  | CRISPR | 48,353 | 48,428 | 75 | 25 | 1 |
|  | CRISPR | 150,234 | 150,317 | 83 | 28 | 1 |
|  | CRISPR | 13,120 | 13,325 | 205 | 23 | 3 |
|  | CRISPR | 249 | 518 | 269 | 27 | 4 |
| *Schaalia odontolytica* NCTC 9935^T^ | CRISPR | 27,846 | 28,007 | 161 | 37 | 1 |
|  | CRISPR | 42,032 | 42,144 | 112 | 30 | 1 |
|  | CRISPR | 73,607 | 73,749 | 142 | 48 | 1 |
|  | CRISPR | 101,899 | 101,973 | 74 | 23 | 1 |
|  | CRISPR | 383,932 | 384,020 | 88 | 28 | 1 |
|  | CRISPR | 395,046 | 395,185 | 139 | 51 | 1 |
|  | CRISPR | 422,708 | 423,831 | 1,123 | 36 | 17 |
|  | CRISPR | 296,707 | 296,794 | 87 | 28 | 1 |
|  | CRISPR | 430,504 | 430,619 | 115 | 34 | 1 |
|  | CRISPR | 2,744 | 3,014 | 270 | 28 | 4 |
| *Schaalia meyeri* DSM 20733^T^ | CRISPR | 31,691 | 31,812 | 121 | 35 | 1 |
|  | CRISPR | 58,335 | 58,423 | 88 | 23 | 1 |
|  | CRISPR | 225,761 | 225,866 | 105 | 28 | 1 |
|  | CRISPR | 276,274 | 278,422 | 2,148 | 36 | 33 |
|  | CRISPR | 43,891 | 44,016 | 125 | 24 | 2 |
|  | CRISPR | 180,835 | 180,913 | 78 | 27 | 1 |
|  | CRISPR | 47,624 | 47,895 | 271 | 29 | 4 |
|  | CRISPR | 180,795 | 180,882 | 87 | 28 | 1 |
|  | CRISPR | 89,623 | 89,717 | 94 | 31 | 1 |

**Table S7. Cas gene distribution of two strains**

| **Strain** | **Element** | **Start** | **End** | **Gene status** | **Cas genes** |
| --- | --- | --- | --- | --- | --- |
| Strain NCTC 9931 | Cas cluster | 61,988 | 64,567 | mandatory | cas3_TypeID |
|  | Cas cluster | 28,129 | 29,091 | mandatory | cas1_TypeIE |
|  | Cas cluster | 26,655 | 27,383 | mandatory | cas5_TypeIE |
|  | Cas cluster | 27,383 | 28,132 | mandatory | cas6_TypeIE |
|  | Cas cluster | 25,534 | 26,658 | mandatory | cas7_TypeIE |
|  | Cas cluster | 23,171 | 24,841 | mandatory | cse1_TypeIE |
|  | Cas cluster | 24,838 | 25,488 | mandatory | cse2_TypeIE |
|  | Cas cluster | 20,181 | 23,174 | forbidden | cas3_TypeI |
|  | Cas cluster | 20,181 | 23,174 | forbidden | cas3_TypeI |
|  | Cas cluster | 26,655 | 27,383 | forbidden | cas5_TypeIE |
|  | Cas cluster | 27,383 | 28,132 | forbidden | cas6_TypeIE |
|  | Cas cluster | 25,534 | 26,658 | forbidden | cas7_TypeIE |
|  | Cas cluster | 23,171 | 24,841 | forbidden | cse1_TypeIE |
|  | Cas cluster | 24,838 | 25,488 | forbidden | cse2_TypeIE |
| *Schaalia odontolytica* NCTC 9935^T^ | Cas cluster | 42,243 | 43,127 | mandatory | cas4_TypeI-II |
